# Abacavir inhibits but does not cause self-reactivity to HLA-B*57:01-restricted EBV specific T cell receptors

**DOI:** 10.1038/s42003-022-03058-9

**Published:** 2022-02-16

**Authors:** Anuradha Sooda, Francois Rwandamuriye, Celestine N. Wanjalla, Lichen Jing, David M. Koelle, Bjoern Peters, Shay Leary, Abha Chopra, Michael A. Calderwood, Simon A. Mallal, Rebecca Pavlos, Mark Watson, Elizabeth J. Phillips, Alec J. Redwood

**Affiliations:** 1 Institute for Immunology and Infectious Diseases, Murdoch University, Murdoch, WA Australia; 2 Telethon Kids Institute, Nedlands, WA Australia; 3 Division of Infectious Diseases, Department of Medicine, Vanderbilt University Medical Center, Nashville, TN USA; 4 Department of Medicine, University of Washington, Seattle, WA USA; 5 Department of Laboratory Medicine and Pathology, University of Washington, Seattle, WA USA; 6 Department of Global Health, University of Washington, Seattle, WA USA; 7 Benaroya Research Institute, Seattle, WA USA; 8 Fred Hutchinson Cancer Research Center, Seattle, WA USA; 9 Division of Vaccine Discovery, La Jolla Institute for Allergy and Immunology, La Jolla, CA USA; 10 Department of Medicine, The Channing Laboratory, Harvard Medical School and Brigham and Women’s Hospital, Boston, MA 02115 USA; 11 Center for Drug Safety & Immunology, Department of Medicine, Vanderbilt University Medical Center, Nashville, TN USA; 12 Institute for Respiratory Health, Level 2, 6 Verdun Street, Nedlands, WA 6009 Australia; 13 School of Biomedical Sciences, University of Western Australia, Perth, WA Australia

**Keywords:** Medical research, Outcomes research

## Abstract

Pre-existing pathogen-specific memory T cell responses can contribute to multiple adverse outcomes including autoimmunity and drug hypersensitivity. How the specificity of the T cell receptor (TCR) is subverted or seconded in many of these diseases remains unclear. Here, we apply abacavir hypersensitivity (AHS) as a model to address this question because the disease is linked to memory T cell responses and the HLA risk allele, HLA-B*57:01, and the initiating insult, abacavir, are known. To investigate the role of pathogen-specific TCR specificity in mediating AHS we performed a genome-wide screen for HLA-B*57:01 restricted T cell responses to Epstein-Barr virus (EBV), one of the most prevalent human pathogens. T cell epitope mapping revealed HLA-B*57:01 restricted responses to 17 EBV open reading frames and identified an epitope encoded by EBNA3C. Using these data, we cloned the dominant TCR for EBNA3C and a previously defined epitope within EBNA3B. TCR specificity to each epitope was confirmed, however, cloned TCRs did not cross-react with abacavir plus self-peptide. Nevertheless, abacavir inhibited TCR interactions with their cognate ligands, demonstrating that TCR specificity may be subverted by a drug molecule. These results provide an experimental road map for future studies addressing the heterologous immune responses of TCRs including T cell mediated adverse drug reactions.

## Abstract (web summary)

HLA-B*57:01 restricted EBV-specific T-cell receptor specificity is altered by abacavir, suggesting a potentially inhibitory effect of abacavir on HLA-B*57:01 restricted TCR recognition.

## Introduction

Pre-existing immunity to pathogens has the capacity to shape host response in a variety of ways. Memory as well as de novo responses to common pathogens are associated with multiple autoimmune diseases^1,2^. Key to understanding these diseases will be understanding the specificity of the responses, through either the B-cell receptor or the T cell receptor (TCR) and how these specificities are subverted or seconded to recognize heterologous antigens.

Abacavir is a nucleoside analog reverse-transcriptase inhibitor, which is used to treat HIV-1 infection in combination with other antiretrovirals. However, abacavir is also associated with a potentially life-threatening reaction known as abacavir hypersensitivity syndrome (AHS). Historically, AHS was diagnosed in 5–9% of treated patients^3^ with symptoms including, fever, malaise, gastrointestinal, and respiratory symptoms, and a relatively late manifestation of mild to moderate skin rash in 70% of cases^4,5^. AHS is restricted to patients carrying HLA-B*57:01 and not other closely related members of the B17 serotype, such as HLA-B*58:01 or HLA-B*57:03^6–9^. While carriage of the HLA-B*57:01 allele provides a 100% negative predictive value, only 55% of carriers develop AHS (55% positive predictive value, PPV)^7^. Structural studies indicate that abacavir binds non-covalently within the floor of the HLA-B*57:01 peptide-binding cleft^10,11^. This alters the peptide-loading complex of HLA-B*57:01, resulting in a preference for smaller aliphatic residues at the C-terminal, such as valine, isoleucine, or leucine, compared to normally preferred aromatic anchor residues, tryptophan or phenylalanine^10,11^. As a result, the repertoire of peptides presented to CD8^+^ T cells is altered in the presence of abacavir and recognized as foreign and capable of eliciting an immune response^10–12^. This altered-self model otherwise known as the altered peptide repertoire model is defined by AHS which helps to explain the unique role of CD8^+^ T cells in this condition.

AHS symptoms can occur within 1.5 days of first drug exposure in cases of AHS that are later found to be patch test positive and^7^ suggest the existence of pre-formed memory responses in affected patients^6^. Moreover, abacavir-responsive CD8^+^ T cells can be identified in the blood of abacavir-naïve, HLA-B*57:01^+^ individuals^6^. Together these data suggest pre-existing memory T cells cross-recognize the tri-molecular complex of HLA-B*57:01, abacavir and self-peptide. This heterologous immunity model provides an ideal opportunity to assess the impact of TCR specificity in adverse immune mediated events, because the initiating insult, abacavir, and the necessary HLA allele, HLA-B*57:01, are known. The identification of the TCR that recognizes both a pathogen and abacavir plus self-peptide would not only provide formal support for the heterologous immunity model and potentially explain the incomplete PPV of HLA-B*57:01 carriage^6,13^ but may also provide a roadmap for other studies seeking to determine how TCR specificity can be subverted to cause disease.

Memory responses to human herpesviruses (HHVs) are likely candidates along the road to the development of AHS. HHV are ubiquitous in the human population and generate a large pool of memory T cells in infected patients. Epstein Barr virus (EBV) is amongst the most prevalent of the HHVs, infecting over 90% of the adult human population and typically by young adulthood in developed countries^14^. CD8^+^ T cells play a central role in controlling EBV infection. EBV-specific CD8^+^ T cells are significantly expanded in acute infectious mononucleosis and can account for between 1 and 50% of the total CD8^+^ T cell population in the peripheral circulation^15^. While these responses decrease in magnitude over time, a substantial proportion of memory CD8^+^ T cells specific for EBV are detected in the blood of all infected individuals, irrespective of the time of primary infection^16^. Finally, EBV- specific T cells are known to mediate alloreactivity in the transplant settings^17–20^. Although TCRs specific for EBV have been identified^21–24^, none are known to be restricted by HLA-B*57:01. Therefore, we conducted a genome-wide mapping study to define HLA-B*57:01 restricted EBV-specific TCRs. This ORFeome approach identified 17 ORFs that encoded antigens restricted by HLA-B*57:01. In this study, QSRGDENRGW, an HLA-B*57:01 restricted epitope, was mapped to EBNA3C, and four previously reported epitopes were re-confirmed. Single-cell TCR sequencing was used to define the antigen specific TCRs for QSRGDENRGW as well as a previously defined EBNA3B (VSFIEFVGWL) epitope. To ensure specificity, the dominant α/β TCR pairs for each epitope were cloned into a plasmid reporter system and transfected into Jurkat cells. Cloned TCRs did not cross-react with abacavir plus self-antigen presented by HLA-B*57:01. However, abacavir inhibited the recognition of cognate ligands by both of the cloned TCRs, suggesting that TCR specificity is modified by abacavir with unknown functional implications. This study defines a roadmap for addressing the TCR cross reactivity in multiple settings including T cell mediated adverse drug reactions.

## Results

### ORFeome-wide screening for CD8^+^ T cell responses identified HLA-B*57:01 restricted antigens

**Fig. 1:**
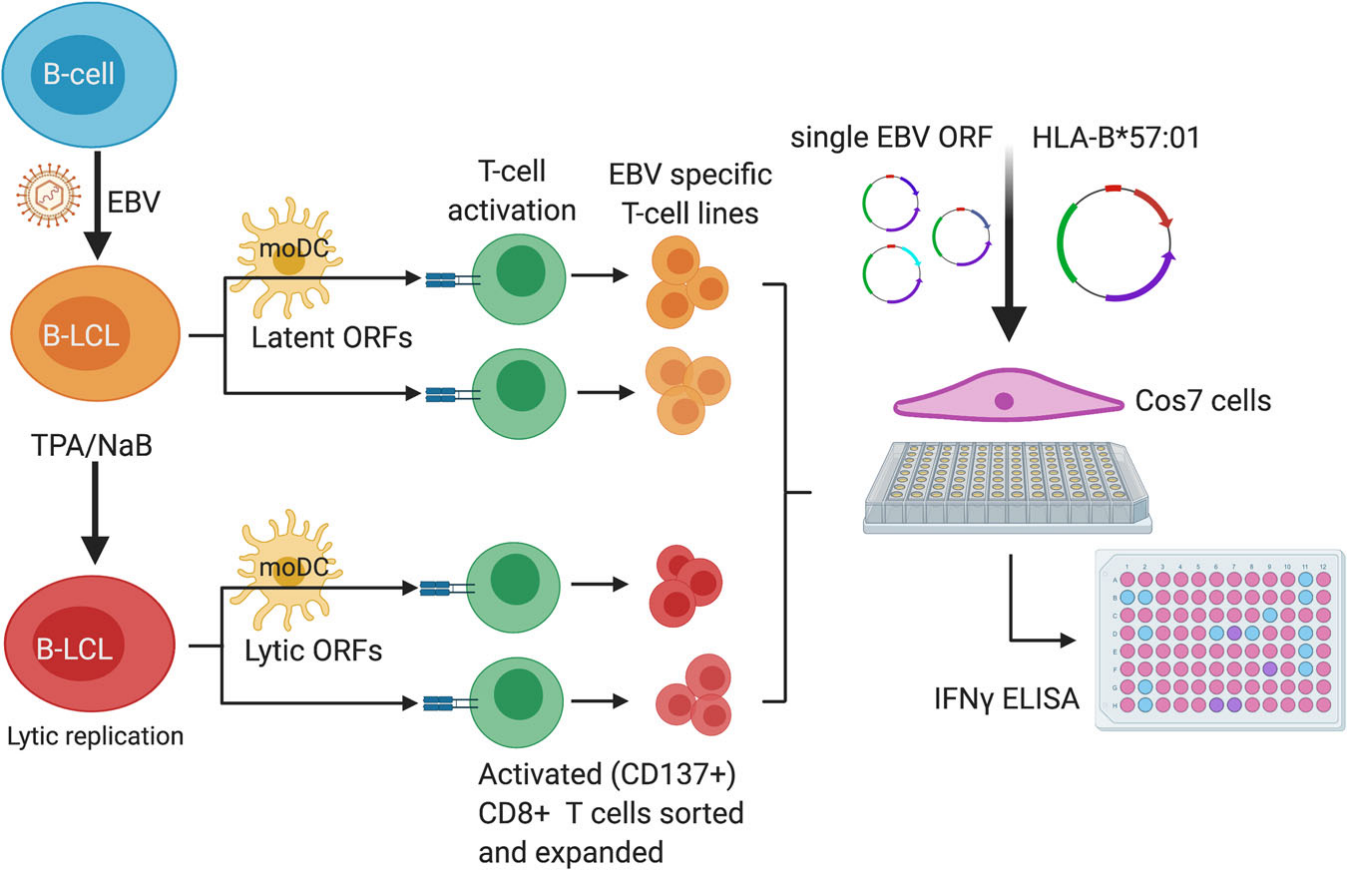
Schematic overview of the production of EBV-specific CD8^+^ T cell lines and their use to define HLA-B*57:01 restricted EBV epitopes. To produce EBV-specific CD8^+^ T cell lines, autologous LCLs were first produced by the infection of B cells with EBV. Autologous LCLs were incubated with donor derived CD8^+^ T cells from PBMCs to identify EBV-specific T cells via the up-regulation of CD137. Flow sorted EBV-specific CD8^+^CD137^+^ T cells were non-specifically expanded by one round of PHA stimulation followed by a second round of anti-CD3. Alternatively, stimulatory LCLs were pre-treated with TPA and NaB to induce lytic replication of EBV and permit the detection of epitopes encoded by lytic ORFs. EBV-specific T cells were also identified in the presence of autologous moDC in LCLs or lytic phase induced LCLs to determine if this allowed for cross presentation and therefore the detection of a broader T cell repertoire. Altogether, four EBV-specific T cell lines were generated from each donor. To determine the EBV specificities of each T cell line, Cos-7 cells were cotransfected with plasmid expressing a single EBV ORF and plasmid expressing HLA-B*57:01. The transfected Cos-7 cells were cultured with each EBV-specific CD8^+^ T cell line for 48 h. HLA-B*57:01 restricted T cell specificity was determined by IFNγ ELISA of the culture supernatant.

To identify HLA-B*57:01 restricted T cells specific to EBV, a method (Fig. 1) for producing and testing EBV-specific cell lines was adapted from Jing et al.^25^. To derive the largest possible pool of EBV epitopes, several means of producing T cell lines were investigated (Supplementary Table 1) as described in the methods. CD8 T cell specificity for individual ORFs was assessed by incubating each T cell line with Cos-7 cells co-transfected with a plasmid encoding HLA-B*57:01 plus a single EBV ORF. All assays were performed in duplicate.

**Fig. 2:**
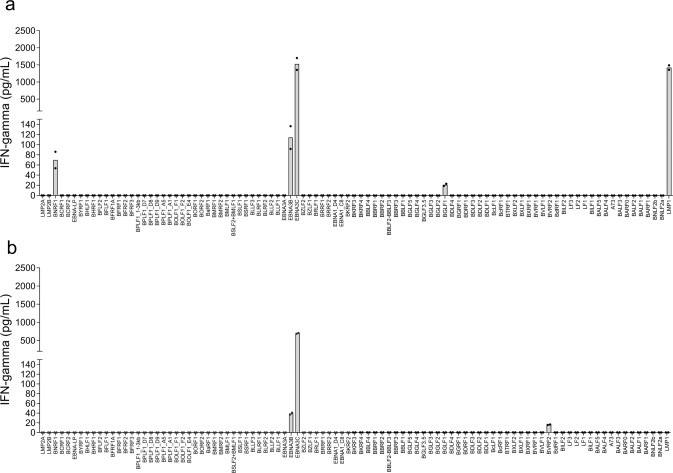
Representative data from donor 2 for IFNγ release by EBV-specific T cell lines stimulated and expanded with LCLs. IFNγ secretion was measured after co-culture of polyclonal CD8^+^ T cells with Cos7 cells individually expressing each EBV ORF in combination with HLA-B*57:01. EBV ORFs are arrayed along the *x*-axis in the order of their location in the EBV genome. The values on the *y*-axis represent IFNγ concentration in pg/mL. IFNγ concentration was extrapolated from a standard curve run with each assay. IFNγ release less than the lowest IFNγ standard used in the study, 12.5 pg/mL, is represented as 0. **a** Mean IFNγ concentration from T_LCL_ following duplicate transfections, and (**b**) Mean IFNγ concentration from T_lyLCL_ following duplicate transfections. Bars represent mean IFNγ concentration; dots are individual data points.

**Fig. 3:**
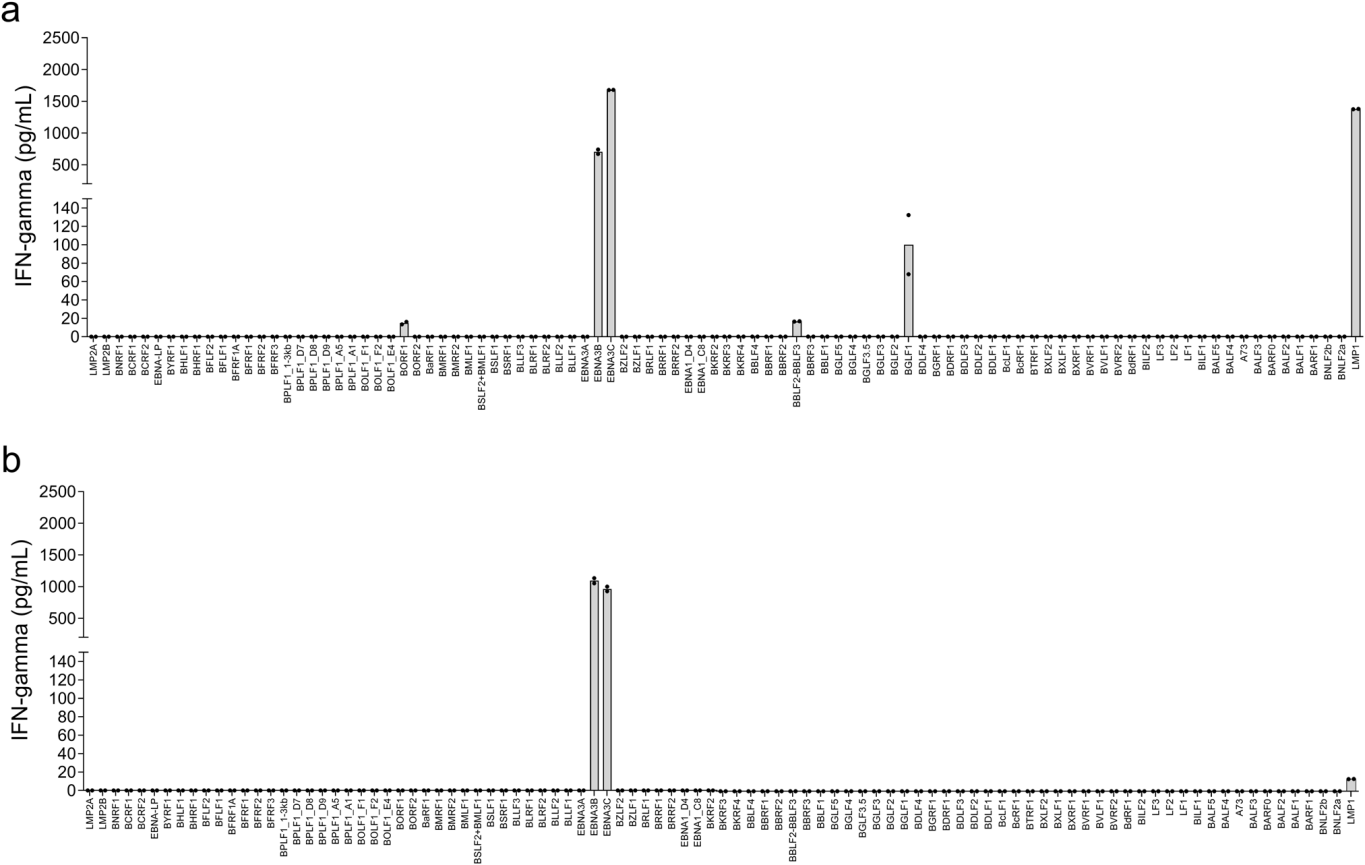
Representative data from donor 2 for IFNγ release by EBV-specific lines stimulated and expanded with moDC plus LCLs. IFNγ secretion was measured after co-culture of polyclonal CD8^+^ T cells with Cos7 cells individually expressing each EBV ORF in combination with HLA-B*57:01. EBV ORFs are arrayed along the *x*-axis in the order of their location in the EBV genome. The values on the *y*-axis represent IFNγ concentration in pg/mL. IFNγ concentration was extrapolated from a standard curve run with each assay. IFNγ release less than the lowest IFNγ standard used in the study, 12.5 pg/mL, is represented as 0. **a** Mean IFNγ concentration from T_DC+LCL_ following duplicate transfection, and (**b**) Mean IFNγ concentration from T_DC+lyLCL_ following duplicate transfection. Bars represent mean IFNγ concentration; dots are individual data points.

Figures 2 and 3 show representative screening data derived from a single donor (Donor 2) using four different T cell lines as outlined in Supplementary Table 1. The CD8^+^ T cell line produced following exposure to primarily latent phase LCLs (T_LCL_) recognized EBNA3B, EBNA3C, LMP1, BGLF1, and BNRF1 (Fig. 2a; Supplementary Data 1). The T cell line produced following exposure to lytic phase LCLs (T_lyLCL_) recognized epitopes present within EBNA3B, EBNA3C, and BVRF2 (Fig. 2b; Supplementary Data 1). T cell lines produced by exposure to LCLs plus DCs (T_DC+LCL_) recognized a broader range of EBV ORF including, EBNA3B, EBNA3C, LMP1, BGLF1, BBLF2-F3, and BORF1 (Fig. 3a; Supplementary Data 1). However, responses to BNRF1, detected using T_LCL_, were lost with T_DC+LCL_. Finally, T cell lines produced in the presence of lytically induced LCLs plus moDCs (T_DC+lyLCL_) recognized only EBNA3B, EBNA3C, and LMP1 (Fig. 3b; Supplementary Data 1).

**Fig. 4:**
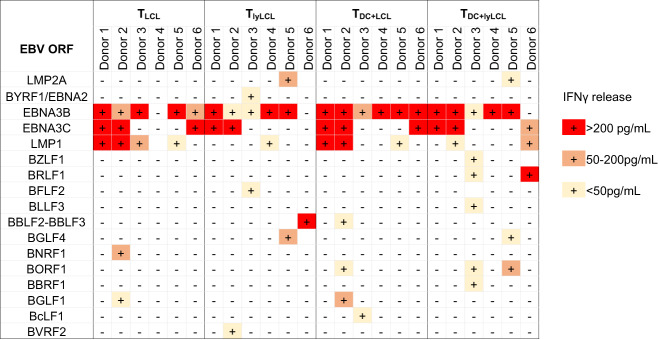
Summary of HLA-B*57:01 restricted responses. Positive ORF targets are arranged vertically in the order of their temporal phase. Individual CD8^+^ T cell responses to targets expanded from four stimulation assays are marked by shaded squares, with red squares identifying prevalent responses (IFNγ > 200 pg/mL), orange squares identifying moderate responses (IFNγ 50–200 pg/mL) and light shade squares identifying weak responses (IFNγ < 50 pg/mL).

Across the six donors (summarized in Fig. 4), HLA-B*57:01 restricted CD8^+^ T cell responses were detected to 17 EBV ORFs. Broadly, T cell lines produced using LCLs alone (T_LCL_) responded to up to five EBV ORFs. Most donors, with the exception of donor 4, responded strongly to 3 EBV latent antigens, EBNA3B, EBNA3C, and LMP1. T cell lines produced using lytic phase induced LCLs (T_lyLCL_) responded to up to 9 EBV ORFs across the individual donors. The most prominent responses, based on IFNγ secretion, were directed to EBNA3B, EBNA3C, and BBLF2-BBLF3, followed by LMP2A and BGLF4 with moderate and weak responses directed to epitopes encoded by EBNA2, BFLF2, BVRF2, and LMP1.

The addition of moDC to the T cell activation protocol broadened the repertoire of detectable responses from 5 to 7 and 9 to 10 ORFs for T cells stimulated with conventional and lytic phase induced LCLs, respectively. Robust T cell responses in the T_DC+LCL_ line were directed to EBNA3B, EBNA3C, and LMP1, followed by moderate responses toward BGLF1 and weak responses directed to BORF1, BBLF2-BBLF3, and BcLF1. Finally, robust T cell responses within the T_DC+lyLCL_ lines were directed toward EBNA3B, EBNA3C, and BRLF1, followed by moderate responses toward BORF1 and LMPl, and weak responses toward LMP2A, BLLF3, BZLF1, BBRF1, and BGLF4. It is important to note that the addition of moDC typically allowed the detection of weak responses, suggesting that cross-presentation enhanced the sensitivity of screening.

### An HLA-B*57:01 restricted epitope was defined in EBNA3C

**Fig. 5:**
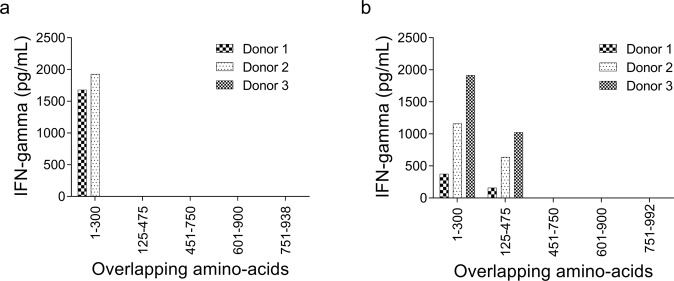
Minigene screening for the identification of an HLA-B*57:01 restricted EBNA3C epitope. **a** EBNA3C and (**b**) EBNA3B full-length ORFs were truncated and cloned into pDEST103 as overlapping minigenes. Minigenes were co-transfected into Cos-7 cells along with HLA-B*57:01. Data are CD8 T cell responses (IFNg production) from the T_LCL_ lines from three donors (Donor 1–3). *x*-axes represent the location of first and last amino acids of the minigene polypeptide sequence.

ORFeome-wide screening of CD8^+^ T cell responses to EBV in healthy donors identified HLA-B*57:01-restricted responses directed predominantly to three latent EBV antigens, EBNA3B, EBNA3C and LMP1. HLA-B*57:01 restricted epitopes within EBNA3C have not been previously defined. To allow EBNA3C epitope mapping, five overlapping EBNA3C minigenes were created (Supplementary Table 2). Likewise, minigenes for EBNA3B were created as a control and to determine if epitopes, other than the previously described VSFIEFVGWL epitope^26^, were also expressed by this ORF. Minigenes were tested as described for full length ORF. For EBNA3C, IFNγ was only detected in response to Cos-7 cells expressing minigene-1, but not with overlapping minigene-2 (Fig. 5a; Supplementary Data 1). Therefore, the EBNA3C epitope must be present within the first 125 amino acids of this protein. Similarly, in EBNA3B, IFNγ production was seen following incubation of T cells with Cos-7 cells transfected with HLA-B*57:01 and with minigenes expressing amino acids 1–300 or amino acids 125–475 (Fig. 5b; Supplementary Data 1). These regions overlap at positions 125–300, corresponding to the known location, at amino acids 278–288, of VSFIEFVGWL^26^.

**Fig. 6:**
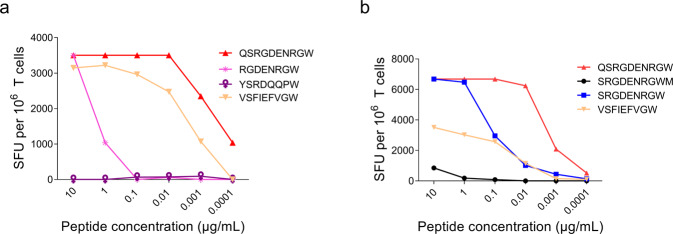
Mapping the HLA-B*57:01 restricted EBNA3C epitope. **a** Identification of the T cell epitope from the EBNA3C 1–300 amino acid minigene. **b** Mapping the minimal QSRGDENRGW epitope. The peptides were screened in an IFNγ ELISpot assay by pulsing K562-B*57:01 with peptides and incubating with T_LCL_ line of donor 1. The data are spot forming units (SFUs) per million cells.

Since the minigene screening experiments suggested that the CD8^+^ T cells recognized an epitope within the first 125 amino acids of EBNA3C, peptides predicted from this region were selected for further screening (Supplementary Table 3). In total, three predicted peptides were synthesized and tested by IFNγ ELISpot (Fig. 6a; Supplementary Data 1). The EBNA3B peptide, VSFIEFVGW, was used as a positive control. Mapping studies identified QSRGDENRGW as the EBNA3C epitope (Fig. 6a; Supplementary Data 1), minimal epitope mapping confirmed that the decamer, QSRGDENRGW (QSR) (Fig. 6b; Supplementary Data 1) was the optimal epitope within EBNA3C.

### QSR-specific responses were abundant in blood of EBV seropositive individuals

**Fig. 7:**
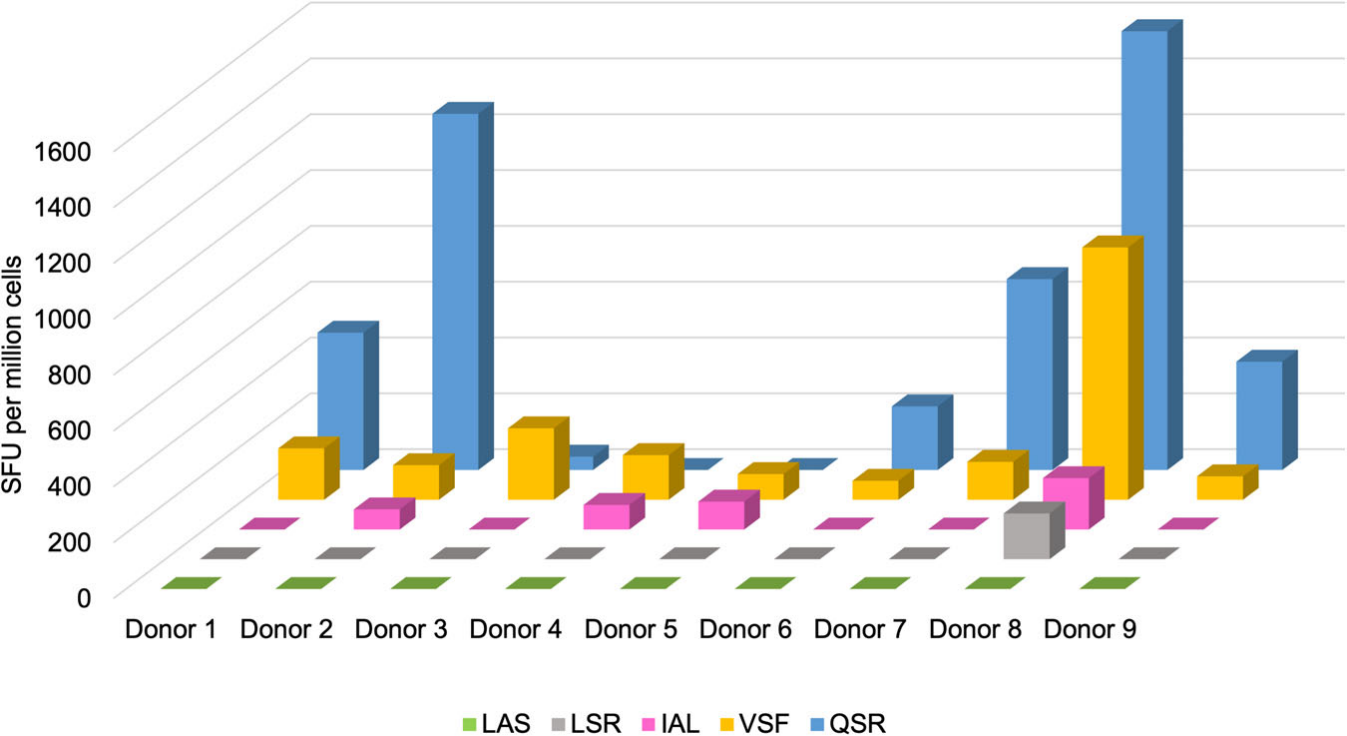
Frequency of T cell responses to HLA-B*57:01 restricted EBV epitopes. The peptides specific to EBNA2 (LASAMRML; LAS), EBNA1 (LSRLPFGMA; LSR), LMP1 (IALYLQQNW; IAL), EBNA3B (VSFIEFVGW; VSF), and EBNA3C (QSRGDENRGW; QSR), were tested by IFNγ ELISpot across six EBV^+^ HLA-B*57:01^+^ donors (Donor 1–6) plus three additional donors (Donor 7–9). T cell responses were tested by pulsing K562-B*57:01 with individual peptides followed by incubation with donor PBMCs. Data are mean spot forming units (SFU) from triplicate wells.

We next compared QSR-specific T cell responses in the blood of EBV seropositive donors to that of previously described HLA-B*57:01-restricted epitopes, EBNA3B (VSFIEFVGW; VSF), LMP1 (IALYLQQNW; IAL), EBNA2 (LASAMRML; LAS), and EBNA1 (LSRLPFGMA; LSR). Data were acquired from the original six HLA-B*57:01 healthy EBV positive donors plus an additional three healthy donors (Supplementary Table 4) by IFNγ ELISpot (Fig. 7; Supplementary Data 1). The majority, 67% (6/9), of individuals responded to the QSR peptide. Responses to the VSF peptide (EBNA3B) were found in all donors, whereas responses to IAL and LSR were identified in 4/9 and 1/9 individuals, respectively. No donor responded to the EBNA1 peptide, LAS. In patients that recognized QSR, this was consistently the dominant response.

### Single cell TCR sequencing revealed antigen specific dominant TCR clonotypes

**Fig. 8:**
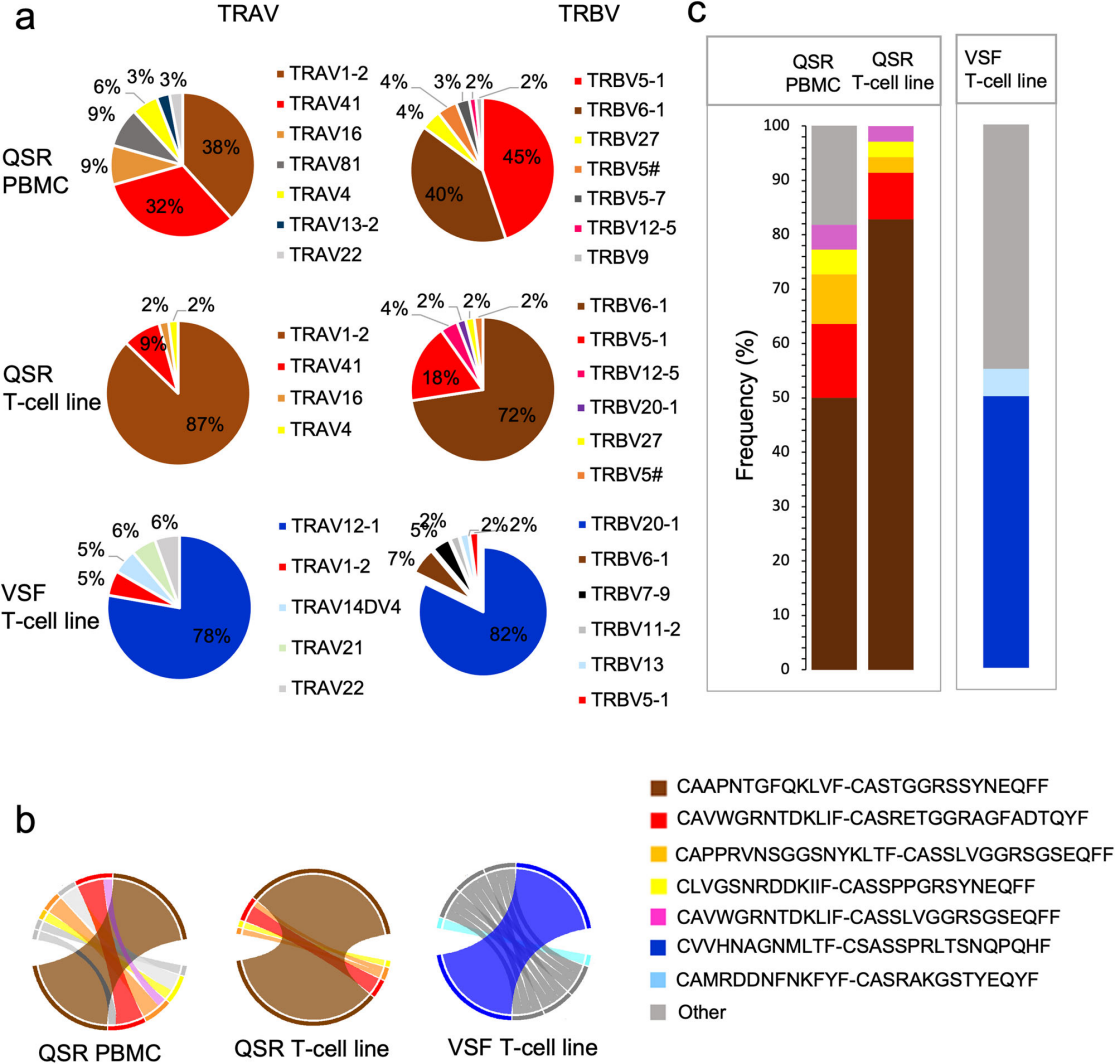
Single-cell TCR repertoire specific to QSR and VSF epitopes from an HLA-B*57:01 donor display dominant TCR clonotypes. QSR PBMC represent single cells specific to QSR isolated from PBMC; QSR T-cell line represent single cells specific to QSR isolated from EBV-specific T cell line; VSF T-cell line represent single cells specific to VSF isolated from EBV-specific T cell line. **a** Each pie chart illustrates the frequency of TCRα and TCRβ sequences of sorted CD8^+^ tetramer specific cells from a single donor (Donor 1). Pie chart colors match clonotypes. **b** Circos plots displaying paired TCR α/β CDR3 combinations in the PBMCs and T cell lines, colors are coded to match that shown in (**c**). The thickness of each segment is proportional to the frequency of the TCR α/β pair. **c** Data from circos plots represented as percentages. Data are color matched to (**b**) where possible, whilst the remaining unique combinations are shown grouped (gray) and marked as other.

To identify the TCRs specific to the two dominant antigens, EBNA3B and EBNA3C, QSR and VSF tetramers were used to sort antigen specific CD8^+^ T cells from T cell lines and PBMCs of donor 1. The QSR-specific CD8^+^ TCRαβ repertoire in the blood (Fig. 8a) was dominated by TRAV1-2 and TRAV41 clonotypes representing 38 and 32% of the total TCRα repertoire, respectively. The TCRβ repertoire was dominated by TRBV5-1 with 45% and TRBV6-1 with 40% of the total TCRβ repertoire. A similar trend was observed in QSR-specific CD8^+^ TCRαβ repertoire in the T cell line (Fig. 8a), where the repertoire was dominated by TRAV1-2 clonotype with 82% and TRAV41 clonotype with 9% of the total TCRα repertoire. Similar to PBMCs, the TCRβ repertoire from T cell line was dominated by TRBV5-1 and TRBV6-1 representing 18 and 72% of the total TCRβ repertoire. The dominant TCR pairing was TRAV1-2/TRBV6-1 in both PBMC and T cell line (Fig. 8b), representing 50 and 83% of collective pairing, respectively (Fig. 8c; Supplementary Data 1). Although skewing of the T cell clonotypes was seen in the T cell line, the dominant TCR remained the same as that directly seen ex vivo in the blood.

The VSF-specific CD8^+^ TCRαβ repertoire (VSF T-cell line) was dominated by TRAV12-1 and TRBV20-1 representing 78 and 82% of the total TCRα and TCRβ repertoire, respectively (Fig. 8a). Paired TCRαβ analysis revealed both the dominant pairs, TRAV12-1 and TRBV20-1 were paired (Fig. 8b) representing 50% of collective pairing (Fig. 8c; Supplementary Data 1). There were insufficient VSF specific T cells present in the PBMCs to allow a comparison with TCR usage within the blood.

### EBV specific TCRs are inhibited by abacavir

To define the specificity of the QSR (TRAV1-2/TRBV6-1) and VSF (TRAV12-1/TRBV20-1) TCRs, a modified NFAT-luciferase reporter system^27^, was developed. Here, a single plasmid (pAR100/pAR101) containing the TCRα, TCRβ genes, CD8 alpha, and NFAT-luciferase reporter was produced (Supplementary Figs. 1, 2). The full length TCRα and TCRβ pair sequences specific for QSR (TRAV1-2/TRBV6-1) and VSF (TRAV12-1/TRBV20-1) epitopes were custom-synthesized and cloned into pAR100. The TCRα, TCRβ and CD8α polypeptides are simultaneously expressed via an EF1α/HTLV promoter by linkage with a porcine teschovirus-1 (P2A) peptide (Supplementary Fig. 3).

**Fig. 9:**
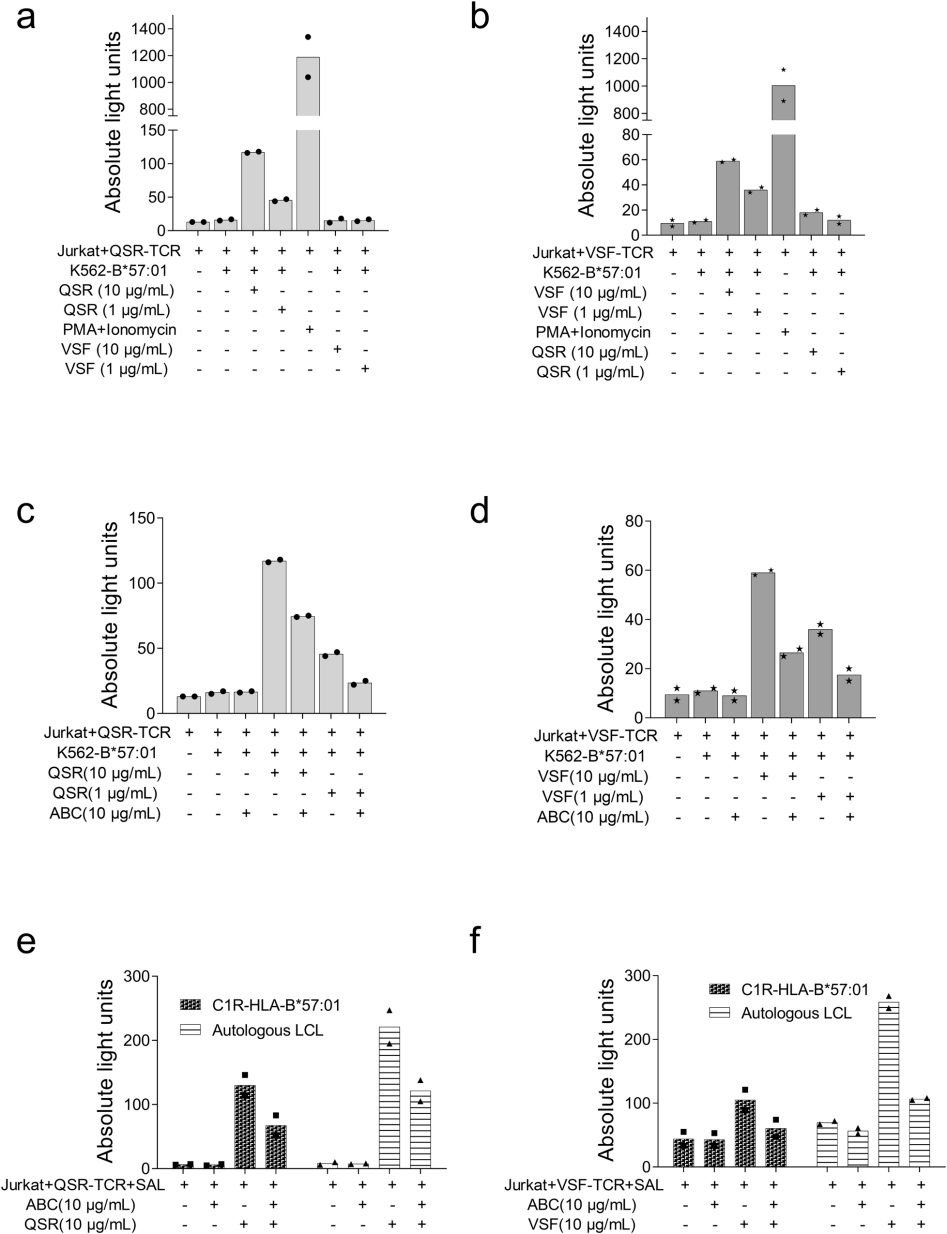
TCR specific for the EBV epitopes, QSR and VSF, recognize cognate ligand but do not cross-react with ABC modified self-peptide. Jurkat cells were transfected with the dominant αβ TCR chains and tested for reactivity to individual peptide epitopes with or without ABC. **a** QSR specific TCR with K562-B*57:01 SAL. **b** VSF specific TCR with K562-B*57:01 SAL. **c** QSR specific TCR with K562-B*57:01 SAL, plus or minus ABC. **d** VSF specific TCR with K562-B*57:01 SAL, plus or minus ABC. **e** QSR specific TCR with alternative APCs (C1R-HLA B*57:01 or autologous LCLs), plus or minus ABC. **f** VSF specific TCR with alternative APC (C1R-HLA B*57:01 or autologous LCLs), plus or minus ABC. PMA-Ionomycin is a Jurkat cell activation control. Luciferase luminescence activity is represented as absolute light units. Bars represent mean values from two replicates; dots are individual data points. Antigen presenting cell (APC) single antigen line (SAL), abacavir (ABC), lymphoblastoid cell lines (LCL).

The single plasmid reporter system was used to first confirm the specificity of each αβ TCR pair and then secondly to determine if either viral-specific TCR could cross-react with abacavir-modified self-peptide. VSF peptide was used as a control for QSR peptide and QSR as a control for the VSF peptide. Plasmids containing either QSR or VSF specific TCRαβ were transiently transfected into Jurkat cells. Engineered Jurkat cells were co-cultured with their cognate peptide-pulsed single antigen cell line (SAL; K562-B*57:01). Specificity via TCR-mediated NFAT signalling was quantified by luciferase luminescence. TCR specificity was confirmed by demonstrating that QSR-specific TCRs recognized QSR peptide-pulsed K562-HLA B*57:01 in a dose-dependent manner, but not VSF-pulsed cells (Fig. 9a; Supplementary Data 1). Likewise, VSF-specific TCRs recognized VSF-pulsed K562-B*57:01 in a dose-dependent manner, but not QSR-pulsed K562-B*57:01 cells (Fig. 9b; Supplementary Data 1).

Having defined the TCRs to recognize EBV epitopes, the hypothesis that EBV-specific TCRs could cross-recognize abacavir modified self-peptide was tested. Jurkat cells transfected with QSR-TCR were co-cultured with abacavir (10 µg/mL) pulsed K562-HLA-B*57:01, C1R-HLA-B*57:01 cells or autologous LCLs. The addition of abacavir alone did not activate QSR-TCR transfected Jurkat cells (Fig. 9c, e; Supplementary Data 1). Likewise, there was no response of VSF specific T cell clones toward abacavir pulsed K562-HLA B*57:01, C1R-HLA-B*57:01 cells and autologous LCLs (Fig. 9d, f; Supplementary Data 1). Furthermore, screening the effects of abacavir on T cell recognition using a polyclonal mix of EBV-specific T cells also demonstrated no cross-reactivity to trimolecular complex of HLA-B*57:01, self-peptide and abacavir (Supplementary Fig. 4a, b). However, notably, pulsing of SAL with peptide plus abacavir negatively impacted T cell recognition, irrespective of the epitope or the TCR (Fig. 9c, d; Supplementary Data 1). In addition, to confirm that the TCR specificity was subverted by abacavir interaction with HLA-B*57:01, we tested TCR interaction by titrating abacavir and including two 6-amino cyclopropyl substituted abacavir analogues, analogue 15 (isopropyl) and 16 (methyl isopropyl)^28^. The abacavir induced down-modulation occurred in a dose-dependent manner (Supplementary Fig. 5a). Interestingly, pulsing of SAL with analogues 15 or 16 plus specific peptide did not impact the specificity of TCR (Supplementary Fig. 5b). Furthermore, to demonstrate that the observed abacavir modulation is not due to non-specific effects on T cell activation, we sought to test using SAL carrying the allelic variant, HLA-B*58:01. The VSF peptide was previously defined as HLA-B*58:01 epitope^26^. We screened VSF-specific dominant TCR identified in this study by co-culturing with peptide ± abacavir pulsed K562-HLA B*58:01 cells. Although the luciferase signal was lower than K562-HLA-B*57:01, Supplementary Fig. 5c shows that the TCR recognition was not affected by the addition of abacavir plus peptide to K562-HLA-B*58:01. These data indicate that a single drug molecule can impact TCR recognition and suggests that non-covalent binding of abacavir to HLA-B*57:01 may dampen specific T cell responses.

## Discussion

Memory T cell responses have been implicated in adverse responses, from transplant rejection to autoimmunity and drug hypersensitivity. However, for many of these interactions the role of specific TCRs remains unclear. AHS provides a powerful model to investigate the means by which TCR specificity can be subverted or co-opted to cause disease because the risk factors, HLA-B*57:01 carriage and the cause of the diseases, abacavir is well understood. Also, the chemistry of abacavir binding to HLA-B*57:01 has been mapped structurally. Although 17 ORFs encoded HLA-B*57:01 restricted epitopes, the predominant focus of the CD8^+^ T cell response was on EBNA3B, EBNA3C, and LMP1. Two epitopes, VSFIEFVGW in EBNA3B and IALYLQQNW in LMP1 have been previously defined^26,29^. An HLA-B*57:01 restricted epitope, QSRGDENRGW, was defined in this study within EBNA3C. Single-cell TCR sequencing of QSR and VSF specific T cells demonstrated a dominant clonotype for each epitope, with multiple sub-dominant TCRs. TCR specificity was confirmed, using a TCR expression system, for each of the dominant TCR clonotypes. For the two antigens assessed, TCR cross-reactivity to abacavir plus self-peptide was not demonstrated. However, abacavir did inhibit the recognition of cognate ligands indicating that this drug can subvert normal TCR recognition.

This is the first report, to our knowledge, of ORFeome-wide mapping of the CD8^+^ T cell responses to HLA-B*57:01 restricted EBV epitopes. An earlier study of CD8^+^ T cell responses to EBV, explored the responses to a panel of 72 lytic phase antigens restricted to a range of HLA alleles, but did not include the abacavir risk allele, HLA-B*57:01^30^. This study provided a complete picture of memory CD8^+^ T cell responses in 6 EBV^+^ HLA-B*57:01^+^ donors to the full panel of 88 EBV ORFs. Among latent proteins, CD8^+^ T cell responses in healthy virus carriers focus predominantly on epitopes within EBNA3A, EBNA3B and EBNA3C, with subdominant responses most frequently recognizing LMP2, less frequently EBNA2, EBNA-LP and LMP1 and very rarely if ever to EBNA1^31,32^. The observations in the present study confirm this pattern of dominance, where T cell responses were predominantly focussed on EBNA3B and 3C. However, contrary to previous studies, responses to LMP1 were observed in 6/6 donors using EBV-specific line stimulated and expanded from at least one of the four approaches used to produce EBV-specific T cell lines (Fig. 4). However, LMP1 responses were only seen in the blood of 3/6 original donors, plus an additional 1/3 HLA-B*57:01 healthy EBV positive donors (Fig. 7), suggesting low precursor frequency for these CD8 T cells.

The CD8^+^ T cell responses to lytic proteins are skewed more to late (L) phase lytic antigens in long-term EBV seropositive individuals compared to those with acute infectious mononucleosis^30^. During long-term carriage, BcLF1 (a major capsid protein) is the most frequent T cell target followed by BZLF1 (immediate early; IE), the early (E) proteins BALF2, BaRF1, BNLF2b and BORF2, and the L proteins BBRF3, BDLF1 and BNRF1^30^. Though the HLA-B*57:01 restricted responses to lytic proteins were typically weak in this study, responses to at least one lytic protein were identified in 4/6 donors. HLA-B*57:01 restricted responses were detected in two IE proteins BZLF1 and BRLF1, four E proteins BFLF2, BLLF3, BBLF3/BBLF3 and BGLF4, and six L proteins BNRF1, BORF1, BBRF1, BGLF1, BcLF1, and BVRF2.

An important caveat in the study of memory T cell responses to pathogens is the precursor frequency of antigen-specific T cells in the blood and the number of PBMC that can be obtained from a donor. To identify the EBV-specific responses that occur at lower frequencies, a large number of T cells with enriched specificity is helpful. Previously, the repertoire of EBV-specific T cells was studied by probing EBV-specific CTL memory with gamma-irradiated autologous LCLs^31,32^, LCLs containing lytically infected cells^33^, and DC plus lytic cell lysate^30^. To our knowledge, no study has compared the T cell response across the entire genome to determine whether the type of pre-stimulation of memory T cells influences antigen detection. In this study, we used four different T cell stimulation methods to generate EBV-specific lines: stimulation with LCLs or lytic phase induced LCLs, or stimulation with LCLs or lytic phase induced LCLs with the addition of moDC. Although using lytically induced LCLs increased the capacity to detect responses to lytic antigens, the responses to some latent antigens such as EBNA3B in donor 6 and LMP1 in donors 1, 2, 3, and 5 were lost. Surprisingly, responses to LMP2A, a latently expressed ORF, were not detected in T cell lines stimulated with conventional LCLs but were seen with T cell lines generated using lytically induced LCLs. This might be due to the modulation of CD8^+^ T cell recognition by LMP2A in latently infected B cells^34^, and the immunomodulatory functions of LMP2A didn’t extend to cells in lytic cycle. However, the mechanisms involved in the recognition of LMP2A in lytic cycle induced LCLs but not in latently infected B cells remains to be investigated. The addition of moDC to LCLs allowed the detection of weak responses to some apparently sub-dominant antigens, however, it resulted in reduced ability to detect responses to other ORFs. For example, in donor 2, the response to BNRF1 seen with the T_LCL_ line was lost in the T_DC+LCL_ line. Similarly, in donor 6, the response to BBLF2-BBLF3 which was detectable using the T_lyLCL_ line, was absent in the T cell line (T_DC+lyLCL_) when moDC were added to the lytic phase induced LCLs (Fig. 4). The reason why responses to specific ORFs were lost with the addition of moDC is not clear and may simply reflect early biases in the selection of CD8^+^CD137^+^ T cells or due to low precursor frequencies of the epitope-specific cells within PBMCs. What is clear is that the addition of moDC, especially given the large number of cells required for production, does not considerably improved genome-wide screening of T cell responses to EBV.

Previous studies have defined the VSFIEFVGW epitope in EBNA3B^26^ and IALYLQQNW in LMP1^29^. This study identified an epitope, QSRGDENRGW, whose optimal length was mapped to amino acid position 60–69 of the nuclear protein, EBNA3C. Robust responses to this epitope with T cell lines were confirmed directly ex vivo from stored PBMCs. For individuals who made a T cell response to the QSR-epitope, the EBNA3C response was dominant relative to EBNA3B, LMP1, EBNA2, and EBNA1.

Single cell TCR sequencing was used to define VSF and QSR TCR usage in a single healthy donor. The TCR repertoire analysis demonstrated a dominant TRAV1-2/TRBV6-1 pair specific to the QSR epitope and TRAV12-1/TRBV20-1 pair specific for the VSF epitope along with a number of subdominant clonotypes. These data are consistent with other studies demonstrating clear hierarchical structures with one or two clonotypes dominating the antigen-specific T cell population^35–37^. TCR clonotypes may become dominant over the course of infection^38^ or during T cell differentiation^39^. The hierarchy of TCR dominance can be preserved over many years for persistent viruses such as EBV^40^ making them attractive targets for the study of heterologous immunity.

A major goal of this study was to determine if an HLA-B*57:01 restricted TCR with confirmed specificity to EBV could cross react with the trimolecular complex of HLA-B*57:01, self-peptide and abacavir. Earlier work provided evidence supporting this hypothesis^6,41^. In this report, we tested two dominant TCRs specific one for QSR (EBNA3C) and the other for VSF (EBNA3B) to provide proof-of-concept that EBV-specific T cells could cross recognize abacavir plus self-peptide. Although the cloned TCRs retained specificity for QSR and VSF respectively, both failed to recognize K562-B*57:01, C1R-B*57:01, or autologous LCLs plus abacavir. Whilst it is possible that the Jurkat cell assay used here was insufficiently sensitive to detect low level abacavir mediated T cell activation, it appears likely that neither of these TCRs is a key driver of the T cell pathology in AHS patients. Carbamazepine induced SJS/TEN has been linked to the carriage of a single TCR^42^, this has not been demonstrated in AHS, and it is possible that pathogenic TCRs are private, and that TCR sequencing and testing will be required from multiple AHS patients and tolerant controls. Furthermore, in support of the altered peptide repertoire model of T cell activation we have previously shown that in abacavir patch test positive skin remote from the original AHS that polyclonal T-effector memory cells are recruited to the site of abacavir exposure^43^.

A surprising finding from this study was that abacavir inhibited the recognition of cognate peptide/MHC by both VSF and QSR specific TCRs. These data suggest a potentially negative effect of abacavir on HLA-B*57:01 restricted TCR recognition. Abacavir shapes the chemistry of the peptide binding groove resulting in a preference for peptides with a C-terminal valine, isoleucine, or leucine in the place of tryptophan or phenylalanine^10,11^. Both peptides tested here have a C-terminal tryptophan and would be subject to abacavir-mediated displacement. However, it is possible that peptide displacement, and altered-self, occurs in all HLA-B*57:01 carriers but only the 55% with endoplasmic reticulum aminopeptidase 1 (ERAP1) allotypes that favor effective peptide trimming experience AHS^44^. It should be noted in this study that peptide was added exogenously and so the role of antigen processing was bypassed. Since abacavir has never been used outside of the setting of HIV it is also not known if this model of AHS might depend on the CD4^+^ T cell depletion that occurs in the setting of HIV^45,46^. If this is the case it is not unreasonable to expect that abacavir would also cause a similar loss of specific activation in vivo that would be most relevant to those tolerating the drug long-term. The clinical relevance of this is unclear, as despite this intriguing hypothesis and although there is no clinical evidence of reactivation of HHVs such as EBV during treatment with abacavir this process could be largely subclinical. Furthermore, the AHS symptoms that occur later than 5 days after drug exposure in 74% of all cases suggests de novo priming of T cells. Evidently, some crucial factors such as danger signals including inflammasome and proinflammatory cytokines are required for priming of T cells. Abacavir mediated NLRP3 inflammasome stimulation implicates potential contribution of innate immune activation in delayed AHS^47^.

In summary, HLA-B*57:01 restricted CD8^+^ T cell responses to EBV, a ubiquitous human herpesvirus, have been established and an immunodominant epitope has been identified. The studies conducted with the single plasmid TCR reporter system developed in this study confirmed that two EBV-specific TCR clonotypes retained specificity for the original EBV epitope. Although, these EBV-specific TCRs were not cross-reactive to self-peptide presented by K562-B*57:01 in the presence of abacavir, the study did show that abacavir could inhibit TCR recognition of cognate ligand. Finally, this study provided an experimental roadmap that can be applied to study heterologous immune responses of other TCRs.

## Methods

### Donor samples

Healthy donors carrying the HLA-B*57:01 allele and seropositive for EBV were recruited at the Australian Bone Marrow Donor Registry. All the donors were HLA typed and PBMCs were separated from heparinised blood samples and stored cryopreserved at the Institute for Immunology and Infectious Diseases (IIID), Murdoch, Perth, Western Australia. The donors used in the present study and their HLA typing is listed in Supplementary Table 4.

### Cell lines and culture

Cos7 cell line was obtained from Department of Microbiology (Royal Perth Hospital, Australia), which were initially obtained from American Type Culture Collection (ATCC, USA). Cos7 cell line was maintained in DMEM media supplemented with 10% fetal bovine serum (FBS) and 1% penicillin-streptomycin at 37 °C and 5% CO2. K562-HLA null parental lines were obtained from Dr. Yvonne Zoet (Leiden University, Netherlands) and K562-B*57:01 single antigen lines were produced in our laboratory by Dr. Dr. Coral-Ann Almeida (IIID, Murdoch University, Australia). Jurkat clone E6-1 was obtained from National Institute of Health (NIH, USA). B95-8 cell line was obtained from Department of Microbiology (Royal Perth Hospital, Australia). Except when stated all the other cell lines were maintained in RPMI-1640 media supplemented with 10% FBS and 1% penicillin-streptomycin at 37 °C and 5% CO2.

### Generation of LCLs from PBMC

EBV-transformed lymphoblastoid B cell lines (LCLs) were generated from cryopreserved donor PBMCs by infection with EBV virus stock^48^. Approximately, 1–3 × 10^6^ PBMCs in 3 mL of RPMI-1640 media supplemented with non-heat inactivated 20% FBS, and 2 µg/mL cyclosporin A (CSA) were infected with 1 mL of EBV stock and cultured for 2–3 weeks. Each week, half of the supernatant was replaced with fresh medium containing 1 µg/mL CSA. Once clusters of cells are visible by light microscopy, cells were continually expanded using fresh media with no CSA until the desired number of cells were obtained.

### Chemical treatment of LCLs

LCLs were seeded at 1 × 10^6^ cells/mL in a six-well plate and treated with 20 ng/mL of TPA and 3 mM of NaB for 24 h to artificially induce the lytic phase^49^. After 24 h, the LCLs were washed three times in RPMI-1640 media supplemented with 10% FBS and used in EBV-specific T cell expansion.

### Preparation of monocyte-derived dendritic cells (moDC)

Monocyte-derived dendritic cells (moDC) were cultured in vitro from cryopreserved PBMCs by plastic adherence in the presence of cytokines, granulocyte-macrophage colony-stimulating factor (GM-CSF) and interleukin-4 (IL-4) for 6–7 days^50^. Approximately, 5–8 × 10^6^ PBMCs were resuspended in 3 mL of RPMI-1640 media supplemented with 1% human serum and 1% FBS and allowed to adhere to the wells of a six-well polystyrene tissue culture plate for 4 h. Non-adherent PBMCs were gently washed off and 6 mL of RPMI-1640 media supplemented with 5% human serum and 5% FBS (T cell media, TCM) containing 10 ng/mL IL-4 and 10 ng/mL GM-CSF was added to each well. After 3 days, the media was replaced with fresh TCM media containing 10 ng/mL IL-4, and 10 ng/mL GM-CSF and incubated for another 3–4 days. After 6–7 days, moDCs were harvested by treating with 20 mM EDTA and cell scraping.

### EBV-specific T cell line production

EBV-specific T cell lines (Supplementary Table 1) were produced from six healthy EBV seropositive donors (Donor 1–6; Supplementary Table 4) by stimulating with autologous LCLs or autologous LCLs induced for lytic EBV replication, plus or minus moDC. For in vitro T cell stimulation through moDC plus LCLs, mature moDCs were resuspended at 1 × 10^6^ cells/mL in TCM and plated at 200 µL/well in a 24-well plate. The seeded moDC were incubated with 200 µL of either irradiated autologous untreated or chemically treated LCLs, which were resuspended at 1 × 10^6^ cells/mL in TCM. For T cell stimulation through untreated or chemically treated LCLs, the irradiated LCLs were seeded at 2 × 10^5^ cells/well in 200 µL of TCM and an extra 200 μL of TCM was added to each well to equalize the volumes with moDC plus LCL assay. Finally, 400 µL of autologous CD8^+^ T cells (2 × 10^6^ cells/well) that were negatively selected using the MACS CD8^+^ T cell isolation kit (Miltenyi Biotec) were added for a responder/stimulator ratio of 10:1 in a final volume of 800 µL and incubated overnight. After stimulation of CD8^+^ T cells for 20 h, the cells were stained with 7-aminoactinomycin D (BD Biosciences, 1:40 dilution), anti-CD3-PE (clone UCHT1, BD Biosciences, 1:100 dilution), anti-CD8-FITC (clone 3B5, Invitrogen, 1:40 dilution), and anti-CD137-Allophycocyanin (clone 4B4-1, BD Biosciences, 1:40 dilution) and sorted for CD8^+^CD137^+^ T cells by gating on live CD3^+^ T cells using a FACSAria II (BD Biosciences). Sorted cells were centrifuged, half of the top media was replaced with fresh media and plated in bulk in a 96-well U-bottom plate. Seeded cells were cultured with 1.5 × 10^5^ allogeneic 3300 rad–irradiated PBMCs and 1.6 μg/ml PHA-P (Sigma-Aldrich) in 200 μL TCM. After 48 h, media was supplemented with natural human IL-2 (32 U/ml, Lonza) and cultured for 2 weeks replacing half of the media with fresh every 2 days. A small portion of the first expansion was further expanded for another 2 weeks by culturing with irradiated allogeneic PBMCs and LCLs supplemented with 30 ng/mL anti-CD3 mAb (OKT3) and recombinant hIL-2 (50 U/mL; PeproTech)^25^.

### EBV ORF cloning

A library or ORFeome representing 85 EBV proteins, was kindly provided by Dr Mike Calderwood (The Channing Laboratory, Harvard Medical School and Brigham and Women’s Hospital, Boston, MA 02115). The ORFs were supplied in a Gateway^®^ entry vector, pDONR223 (Invitrogen) that allows subcloning of ORFs into other expression vectors by Gateway^®^ methods. The three ORFs, BCRF2/BWRF1 (homologous proteins), A73 and BVLF1 were PCR-amplified from B95-8 cell line cDNA template and ligated into pDONR201 Gateway^®^ entry vector. To reflect the unique amino acid content, repeated domains have been deleted from EBNA-LP and LF3 and reduced the size of the ORF. The long ORF, BPLF1 was truncated into six fragments overlapping with several nucleotides. Similarly, BOLF1 was truncated into three overlapping fragments. All the remaining ORFs were cloned as full length except EBNA1. In the case of EBNA1, two small plasmids including amino acids 1–89 and 379–641 were created and the glycine-alanine repeat region from amino acid 90–378 was excluded from the ORF. The EBV ORF library representing 88 individual EBV ORFs was then subcloned into a custom transient expression vector, pDEST103, allowing N-terminal fusion of each ORF to enhanced green fluorescent protein^25^. A full list of 88 ORFs and their size in the constructs is shown in Supplementary Data 2.

### EBV-specific T cell screening

CD8^+^ T cell responses restricted by HLA-B*57:01 were screened using co-transfected Cos-7 cells^51^. Briefly, Cos-7 cells were plated at 1 × 10^4^ cells/well in 96-well flat bottom plates in 200 μL of DMEM. After 24 h, Cos-7 cells were co-transfected with 75 ng of HLA-B*57:01 plasmid DNA and 150 ng of EBV-ORF expression plasmid per well using FuGENE6 (Promega). Each EBV ORF was tested in duplicate transfection experiments. Cos-7 cells transfected with a pDEST103 empty plasmid was used as a negative control. After 72 h, Cos-7 cell media was removed and polyclonal EBV-specific CD8^+^ T cells were added at 1 × 10^5^ cells/well in 200 μL of fresh TCM. After 48 h, supernatants were collected, and T cell activation was identified by IFNγ ELISA.

#### ELISA

T cell activation was detected by supernatant ELISA for IFNγ^51^. Briefly, clear flat-bottom 96-well Maxisorp Nunc-immuno nonsterile plates (ThermoFisher Scientific) were coated with 100 μL of 1 μg/mL of mouse IgG1 recombinant human IFNγ (2G1) diluted in 0.1 M sodium carbonate (pH 9.6) buffer overnight at 4 °C and blocked with 1% BSA in 0.2 M NaCl, 3 mM KCl, 0.05 M Tris, pH 9 (TBS) for 1 h at room temperature. All the washes were performed 3–5 times with PBS/0.2% Tween 20 at room temperature. After blocking, 100 μL of supernatants from EBV ORFeome screening were added. For standards, human recombinant IFNγ ELISA standard serially diluted from 800 to 12.5 pg/mL in TBS with 0.1% BSA, 0.05% Tween 20, and 4 μg/ml Ig-Inhibiting Reagent (Bioreclamation; sample buffer) were added and incubated for 2 h with shaking at room temperature. After washing, 100 μL of mouse IgG1 recombinant human IFNγ biotin conjugate (B133.5), diluted to 100 ng/mL in sample buffer was added for 1 h with shaking at room temperature. After five washes, 100 μL of streptavidin-horseradish peroxide, diluted to 100 ng/ml in TBS with 1% BSA, 0.05% Tween 20 was added for 30 min. Later, ready to use 3,3′,5,5′-TMB substrate was added for 10 min, and the reactions were stopped with 1 M phosphoric acid. The optical density was read at 450 nm wavelength using a DTX 880 multimode detector (Beckman Coulter). Standard curves were constructed using GraphPad Prism^®^ Version 9.1.0 Software (GraphPad Software Inc.). Standard curves were created from serial dilutions of the human recombinant IFNγ ELISA standard starting from 800 to 12.5 pg/mL and subtracting the mean absorbance value of the blank wells. Sample concentrations were calculated using the standard curves in GraphPad Prism^®^ Version 9.1.0 Software (GraphPad Software Inc.).

#### ELISPOT assay

PBMCs or expanded EBV-specific T cells were screened for reactivity to EBV peptides by IFNγ ELISPOT. For minimal/optimal epitopes screening in EBNA3C, the peptides were synthesized with >80% purity and tested using K562-B*57:01 as APC. Polyclonal CD8^+^ T cells (responder cells) and K562-HLA-B*57:01 single antigen cell lines (stimulator cells) were rested overnight and were added to duplicate wells at a responder/stimulator cell ratio of 10:1. The responder/stimulator cells with no peptide stimulation but with 0.1% DMSO were also included in duplicate wells as negative controls. The spots were counted with an automated imaging device, AID ELISpot analyzer (AID GmbH) after air drying the plate overnight. Results from IFNγ-ELISpot assays were expressed as SFU per 10^6^ cells after subtracting the average background counts from unstimulated negative controls. Peptides with more than 50 SFU per 10^6^ PBMCs after subtracting the average background were considered positive.

### Tetramer production

Peptide-HLA class I tetramers were made as previously described^52^. Briefly, 36 µM of biotinylated HLA-B*57:01 molecules were diluted 100-fold into a reaction buffer containing Tris/maleate pH 6.8, 0.03% Lutrol^®^ F68, protease inhibitor mix, recombinant human beta-2 microglobulin and HPLC purified peptides (>80% purity). The reaction mix was incubated at 18 °C for 48 h allowing for folding and complex formation. Then fluorescence labelled streptavidin was added sequentially in three aliquots over 45 min at 4:1 (peptide/HLA complex: Streptavidin) molar ratio to effect tetramerization.

### Tetramer staining and single cell sorting

Approximately 5 × 10^6^ CD8^+^ T cells and 10 × 10^6^ PBMCs were rested overnight and washed twice in PBS. The cells were then supplemented with 0.05 U/µL of RNase inhibitor (RNase out^TM^ recombinant ribonuclease inhibitor) and stained with either PE-conjugated HLA B*57:01/ VSFIEFVGWL or HLA B*57:01/QSRGDENRGW tetramer at a final concentration of 10 nM for 20 min at room temperature in the dark. The cells were pelleted by centrifugation at 400 *g* for 7 min and washed in FACS buffer (PBS + 1%FBS), followed by surface staining with antibodies, fixable viability stain 620 (BD Biosciences, 1:1000 dilution), CD3-AF700 (clone UCHT1, BD Biosciences, 1:50 dilution), and anti-CD8a Allophycocyanin-Fire 750 (clone RPA-T8, BioLegend, 1:40 dilution) for 30 min at room temperature in the dark. After washing the cells twice in FACS buffer, cells were resuspended in 500 µL of FACS buffer and strained using a polystyrene tube with cell strainer cap (In Vitro Technologies) and supplemented with 0.1 U/µL RNase inhibitor. Cells were left on wet ice and protected from light until the time of sort. Finally, individual cells were sorted directly into each well of a 96-well plate containing 3 µL of lysis buffer using the FACSAria III cell sorter.

### TCR sequencing from single cells

TCR sequencing from single cells was carried out at IIID, Murdoch University. In brief, the single cells were reverse transcribed into cDNA, during which individual cell’s cDNA products were barcoded and generically tagged with both 3′ oligo-dT–and 5′ biotin–labelled template switching oligonucleotides. The cDNA of each cell was subsequently targeted with a combination of nested generic tags and suitable gene-specific primers (e.g., TCRα or TCRβ conserved regions). Samples were multiplexed for next-generation library preparation and sequencing. The TCR sequencing data was analyzed using an in-house software package, Visual Genome Analysis Studio^53^.

### pAR100 and pAR101 cloning

To create pAR100, CD8a was PCR-amplified from pORF9-hCD8Aa (InvivoGen) plasmid using CD8_F and CD8_R primers flanking with *AgeI* and *AflII* restriction enzyme sites (Supplementary Table 5), respectively. The 1.3 kb PCR fragment was digested with *AgeI* and *AflII* and cloned into a similarly digested pSELECT-GFPzeo-mcs plasmid (InvivoGen). The resultant plasmid, pIIID_GFP_CD8, was linearized with *NheI*, dephosphorylated with calf intestinal alkaline phosphatase and a multiple cloning site (MCS) I was added. To generate MCSI, forward and reverse primers containing *AflII*, *ApaI*, *AscI*, *BsiWI*, *BstZ17I*, *NruI*, *PstI,* and *SacII* flanking with *NheI* overhangs were designed and annealed. For annealing, 20 µM of each primer was mixed in the presence of NEB Buffer#2 (New England Biolabs) and annealed at 95 °C for 5 min followed by cooling to room temperature. The resultant annealed MCSI with *NheI* overhangs was ligated into *NheI*-linearized pIIID_GFP_CD8 plasmid to produce pIIID_GFP_CD8_MCSI. Similarly, an additional MCS, MCSII, containing *KpnI*, *XbaI*, *BamHI* and *SalI* flanking with *EcoRI* overhangs was designed, annealed and ligated into *EcoRI*-linearized pIIID_GFP_CD8_MCSI plasmid to produce pIIID_GFP_CD8_MCSI_MCSII. Later, luciferase with the NFAT promoter from pGL3- NFAT luciferase plasmid (Addgene) was digested with *KpnI* and *SalI* and ligated into a similarly digested pIIID_GFP_CD8_MCSI_MCSII plasmid. The resultant plasmid was named pAR100 (Supplementary Fig. 1; Addgene #180377).

To create pAR101 which allows Gateway^®^ cloning of TCRs, reading frame cassette A was PCR-amplified from pDONR201 (Invitrogen) using attR1-KpnI and attR2-EcoRV primers flanking with *KpnI* and *EcoRV*, respectively. The PCR amplified reading frame cassette A was digested with *KpnI* and *EcoRV* and ligated into similarly digested pcDNA3.1(+) plasmid (Invitrogen). The resultant plasmid, pcDNA3.1-attR1R2 was digested with *AflII* and *ApaI* and ligated into similarly digested pAR100. The final plasmid was named pAR101 (Supplementary Fig. 2; Addgene #180378). Both the plasmid maps were confirmed by Sanger sequencing. All the primers used to construct the plasmids were listed in Supplementary Table 5.

### TCR-α and TCR-β chain gene synthesis and cloning

The full length TCR-α and TCR-β chain genes were custom-synthesized with no codon-optimization by Integrated DNA Technologies (USA) in pUCIDT-Amp vector. The synthesis included the restriction enzyme, *AflII* and a self-cleaving peptide, porcine teschovirus-1 (P2A) at the 5′ end followed by TCR-α and TCR-β chain genes linked together with another P2A, and *SacII* at the 3′ end. The defined epitope-specific TCR-α and TCR-β chain genes linked with P2A in pUCIDT-Amp plasmid were excised with *AflII* and *SacII* and ligated into a similarly cut pAR100 plasmid.

### Transfection of effector Jurkat cells

Jurkat cells were cultured and passaged at 0.5 × 10^6^ cells/mL at least once before transfection. The cells were transfected when they were in log phase growth. For transfection, cells were pelleted and washed once in Opti-MEM^™^ reduced serum media and resuspended at 5 × 10^7^ cells/mL in OptiMEM^™^. Transfection of Jurkat cells was performed by electroporation. A total of 1 × 10^7^ cells in 200 μL per experiment were transferred to a 0.4 cm cuvette (Bio-Rad Laboratories, Inc.) containing 10 μg of pAR100 plasmid and electroporated using a Gene Pulser Xcell^™^ Electroporation System (Bio-Rad Laboratories, Inc.) using the square-wave protocol: SQR wave, 230 V, 25 ms, 1 pulse with CE module (V, % droop). After 10 min of recovery at room temperature, cells were transferred to a six-well plate containing 5.8 mL of RPMI-1640 with no phenol red and incubated 24 h prior to co-culture. Transfection efficiency was assessed by flow cytometry to detect GFP and surface expression of CD8A with anti-CD8a Allophycocyanin-Fire 750 (clone RPA-T8, BioLegend, 1:40 dilution) (Supplementary Fig. 6a, b).

### Co-culture and luciferase reporter assay

Luciferase reporter assay was performed as described^54^. Briefly, for peptide and/or drug pulsing targets, K562-B*57:01 single antigen lines were resuspended at 1 × 10^6^ cells/mL in R10 (no phenol red), and 50,000 cells were added to each well of a U-bottom 96-well plate. Either 10 μg/mL or 1 μg/mL of peptide and/or 10 μg/mL of abacavir were added to the corresponding wells and cells were incubated at 37 °C and 5% CO2 for 20–24 h. The final volume in each well was adjusted to 70 μL with R10 (no phenol red). After 24 h, Jurkat effector cells were resuspended at 1.65 × 10^6^ cells/mL and 50,000 cells (30 μL) were added to target cells, yielding a responder to APC ratio of 1:1 in a final volume of 100 µL. The cells were incubated at 37 °C in 5% CO2 for 6 h. After incubation, the entire volume of Jurkat-K562-B*57:01 co-culture was transferred to a white Cliniplate (ThermoScientific) and 100 μL of Luciferin BrightGlo or Steady-Glo was added to each well. After incubating the plates in the dark for 10 min at room temperature, luciferase activity was measured on a DTX 880 multimode detector (Beckman Coulter) using 3000 ms integration. PMA/Ionomycin was used as a positive control. No stimulation negative controls including media only, Jurkat only, and Jurkat with K562-B*57:01 were included in each experiment.

### Study approval

Ethics approval for the conduct of this research project was obtained from Murdoch University Human Research Ethics Committee (2017/246). All the participants provided informed written consent.

### Statistics and reproducibility

In keeping with editorial guidelines, statistical analysis was not performed on experimental replicates, no other data required statistical analysis. Graphical representations were carried out using GraphPad Prism, version 9.1.0 (GraphPad Software Inc.) or Visual Genome Analysis Studio for TCR sequencing studies. In bar graphs, bars represent mean of replicate data, individual data points are shown as dots.

### Reporting summary

Further information on research design is available in the Nature Research Reporting Summary linked to this article.

## Supplementary information

Supplementary Information

Supplementary Data 1

Supplementary Data 2

Reporting Summary

## Data Availability

Raw TCR sequencing data are available on the SRA database, accession number PRJNA706784. Source data used for the generation of Figs. 2, 3, 5, 6, 7, 8c and 9 are provided in Supplementary Data 1. Supplementary Data 2 contains a list of 88 ORFs used in the study and their size in the constructs.
