# Supplementary material for: Abacavir inhibits but does not cause self-reactivity to HLA-B*57:01-restricted EBV specific T cell receptors
Source: Commun Biol. 2022 Feb 16;5:133. doi: 10.1038/s42003-022-03058-9 (PMC8850454; doi:10.1038/s42003-022-03058-9)
Supplement: Supplementary file 1 — Supplementary Information [file 42003_2022_3058_MOESM1_ESM.pdf]

Supplementary information

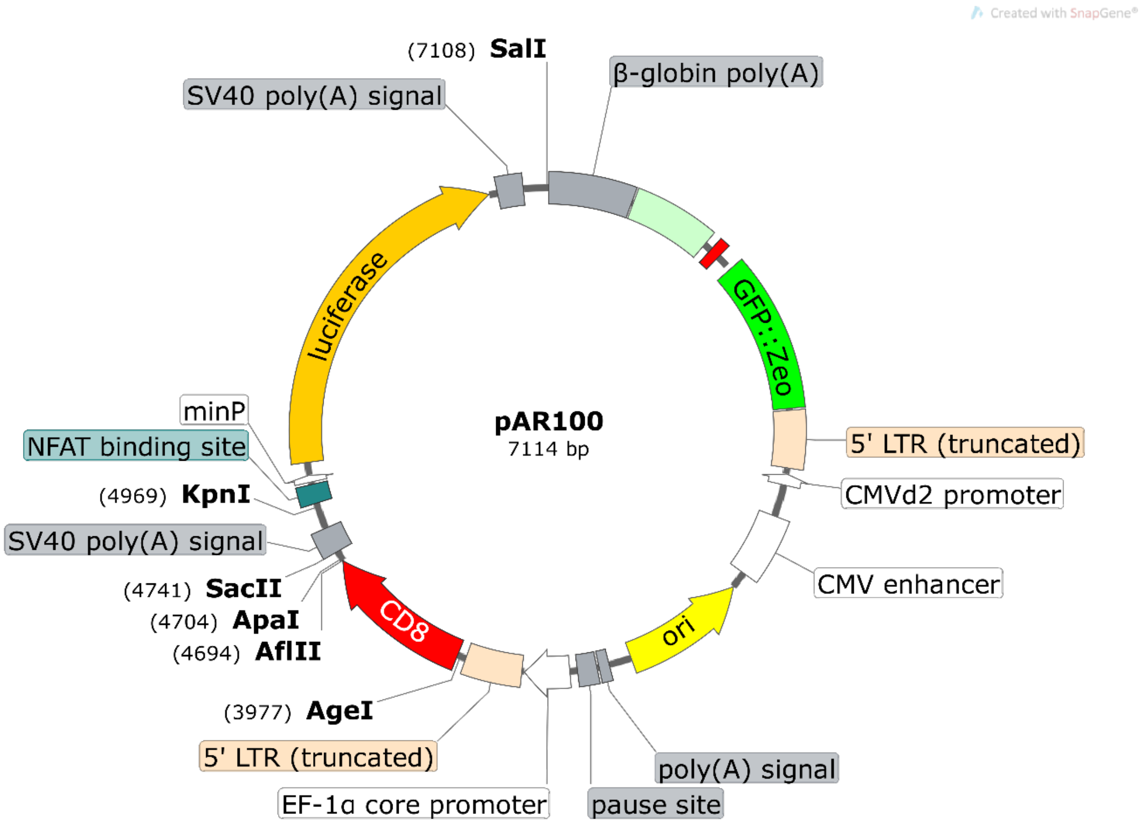

Supplementary Fig. 1. pAR100 allows cloning of TCRα and TCRβ genes using restriction enzymes (*AflII*, *ApaI*, and *SacII*). Composite promoter comprising the Elongation Factor-1α (EF-1α) core promoter and 5' untranslated region of Human T-cell Leukemia Virus (HTLV) drives the expression of CD8, cloned TCRα and TCRβ genes simultaneously. This is combined with NFAT binding sequence upstream of a luciferase reporter, which can be used to assay signal transduction through TCR-induced calcium flux. GFP::zeo is a fusion gene that encodes GFP and resistance to Zeocin™ in mammalian cells, which can be used to monitor transfection efficiency.

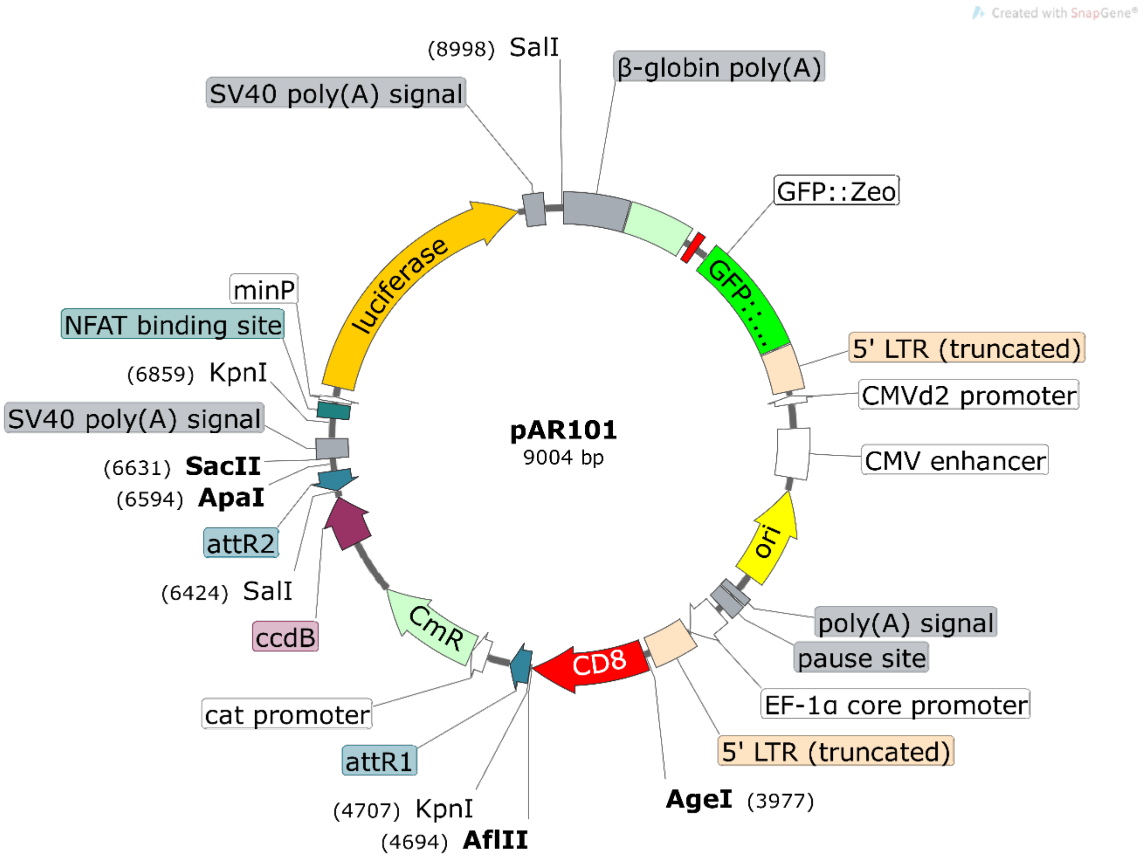

Supplementary Fig. 2. PAR101 plasmid allows cloning of TCRα and TCRβ genes using Gateway® Cloning. Composite promoter comprising the Elongation Factor-1α (EF-1α) core promoter and 5' untranslated region of Human T-cell Leukemia Virus (HTLV) drives the expression of CD8, cloned TCRα and TCRβ genes simultaneously. This is combined with NFAT binding sequence upstream of a luciferase reporter, which can be used to assay signal transduction through TCR-induced calcium flux. GFP::zeo is a fusion gene that encodes GFP and resistance to Zeocin™ in mammalian cells, which can be used to monitor transfection efficiency. Gateway® attR sites allow site-specific and directional recombination cloning of TCRα and TCRβ genes.

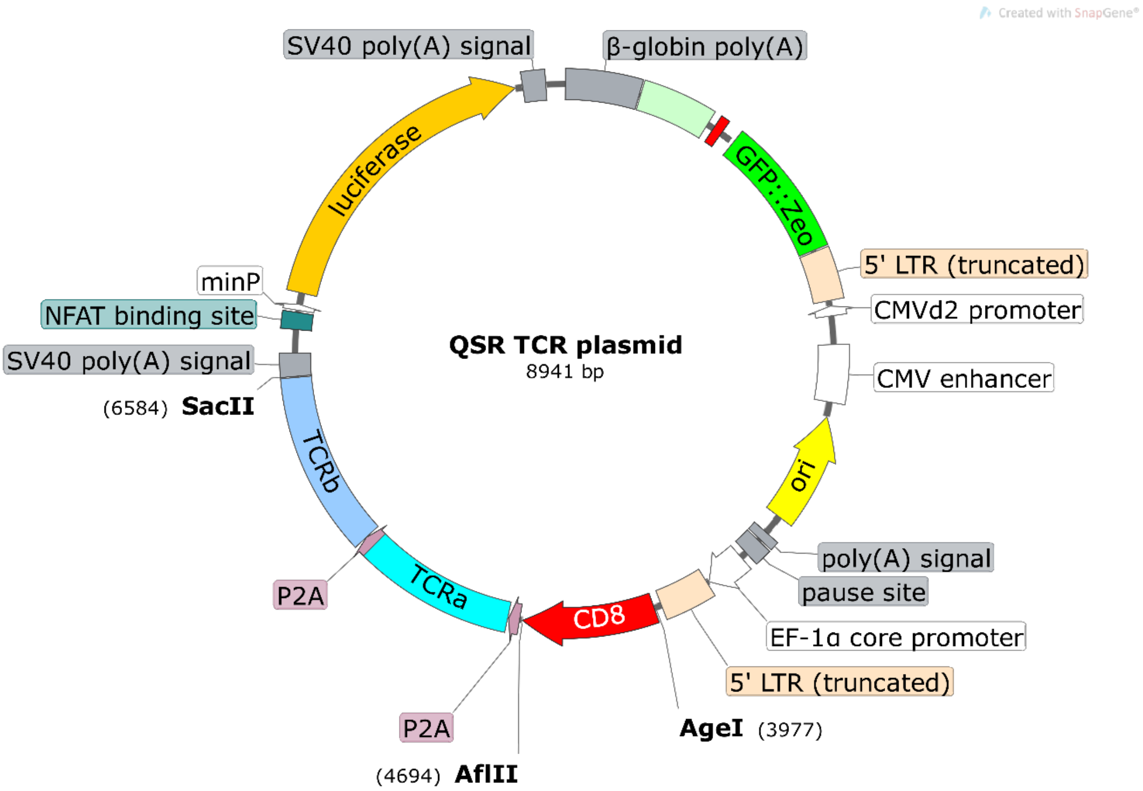

Supplementary Fig. 3. QSR specific TCRα and TCRβ sequences cloned into pAR100 construct and CD8α are simultaneously expressed via EF1α/HTLV promoter by linking with porcine teschovirus-1 (P2A) peptide.

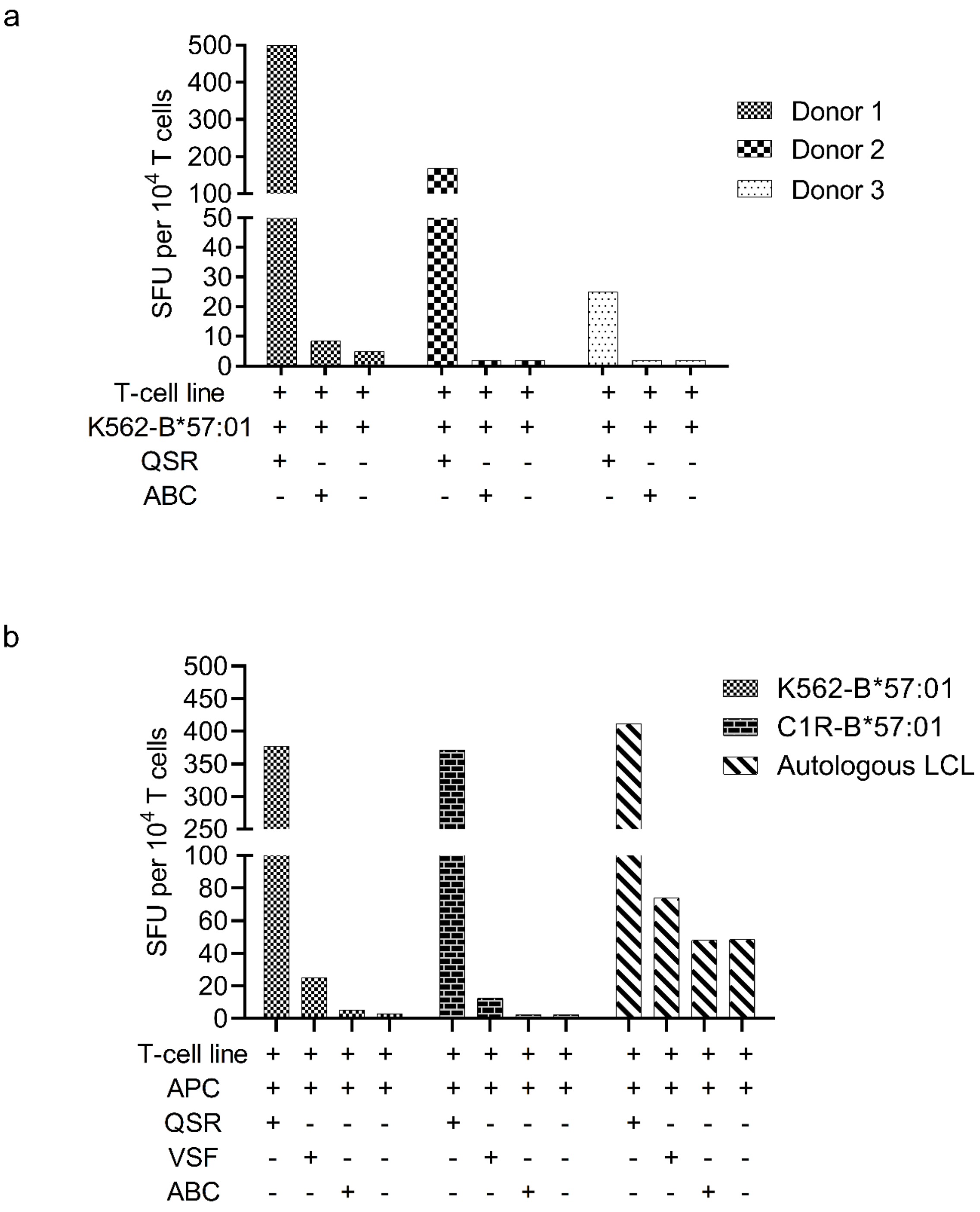

Supplementary Fig. 4. IFN-γ ELISpot assay with EBV-specific polyclonal T-cell lines confirms lack of ABC cross-reactivity. **(a)** The QSR, VSF peptides and ABC were tested at 10 µg/mL concentration across three EBV^{+} HLA-B*57:01^{+} donors (Donor 1-3). Screening was performed by pulsing K562-B*57:01 with peptides or ABC and incubating with donor T-cell lines. **(b)** The EBV-specific T-cell lines were tested for cross reactivity with ABC modified self-peptide presented by various APCs, K562-B*57:01, C1R-B*57:01, and autologous donor LCL. APC+T-cell line without peptide or ABC was used as a negative control. Data are average SFU from duplicate wells. Peptide and ABC were used at 10 µg/mL. Abbreviations: antigen presenting line (APC); abacavir (ABC); spot forming units (SFU).

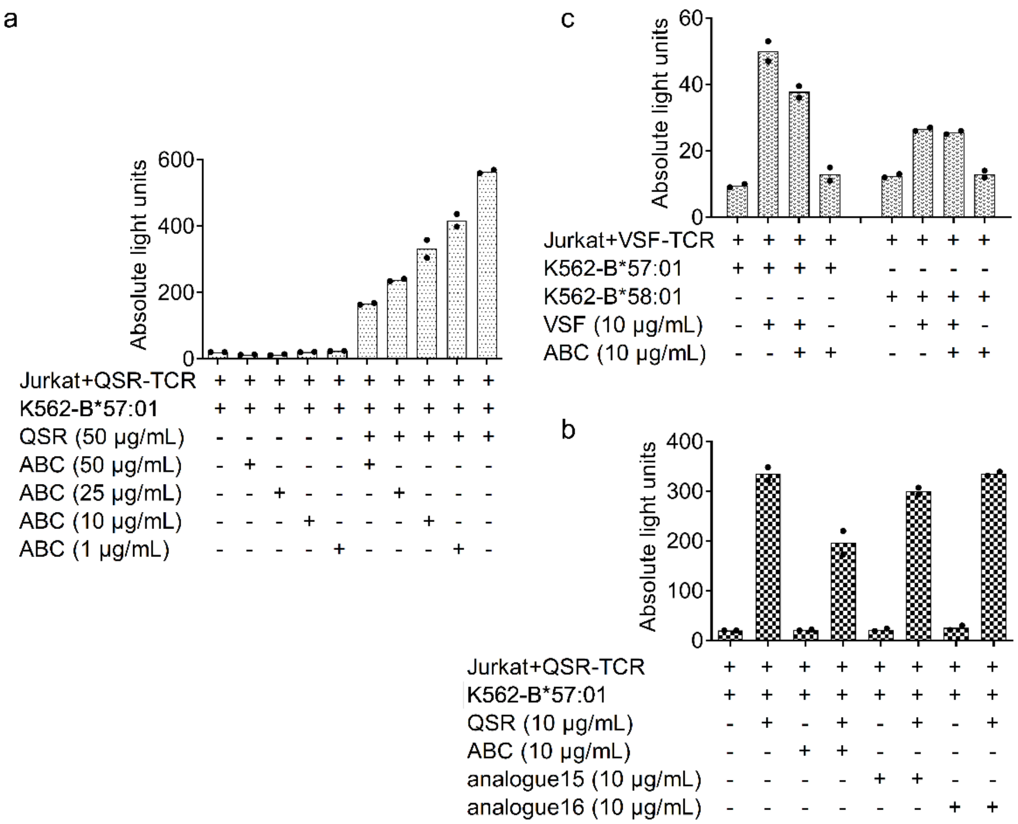

Supplementary Fig. 5. TCR specificities were subverted by abacavir. (a) Engineered Jurkat cells specific for QSR-TCR were tested by co-culturing with K562-B*57:01 SAL pulsed with ABC concentrations ranging from 50 µg/mL to 1 µg/mL. **(b)** The effects of ABC on TCR specificity were validated by including two ABC analogues, analogue15, and analogue16 as controls. Engineered Jurkat cells specific for QSR-TCR were co-cultured with K562-B*57:01 SAL pulsed with ABC or analogues. **(c)** The ABC modulation on T-cell activation was further analysed by co-culturing of engineered Jurkat cells specific for VSF-TCR with ABC pulsed SAL carrying the allelic variant, HLA-B*58:01. Luciferase luminescence activity was represented as absolute light units. Bars represent mean from 2 replicates; dots are individual data points; Abbreviations: single antigen line (SAL); abacavir (ABC).

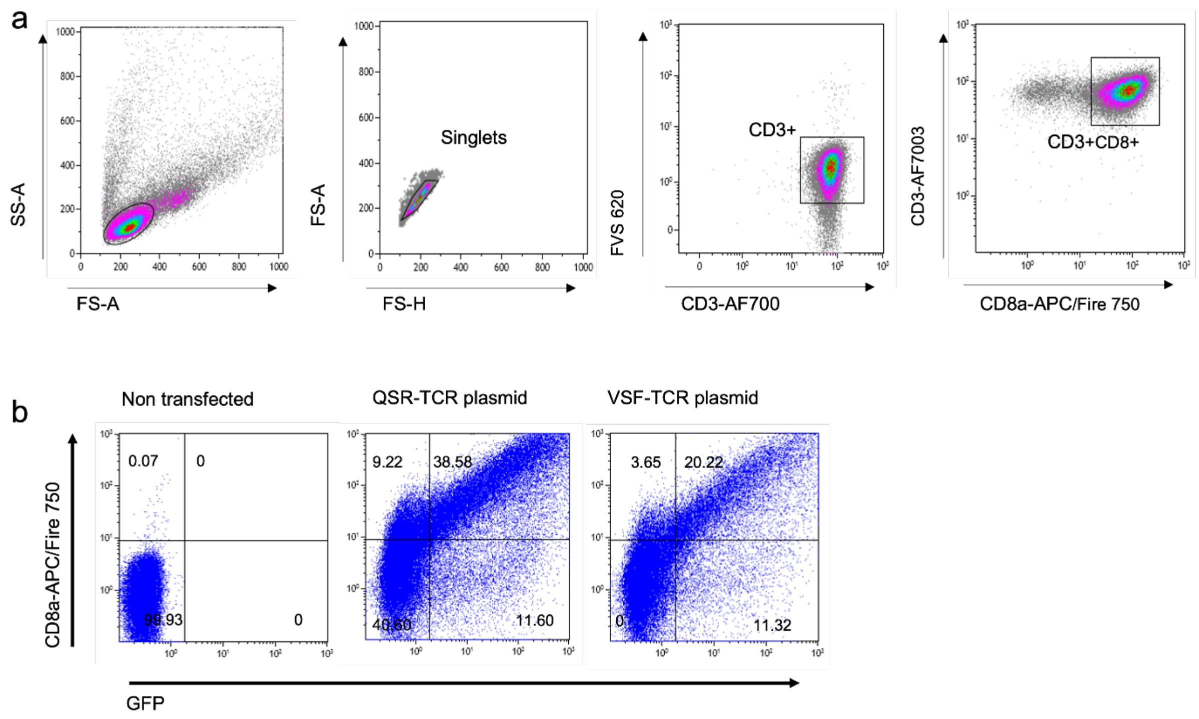

Supplementary Fig. 6. Flow cytometric determination of transfection efficiency based on GFP and CD8 expression in Jurkat cells. a) Gating strategy b) Flow plots illustrate expression of GFP and CD8α detected in non-transfected cells, cells transfected with QSR-TCR plasmid and cells transfected with VSF-TCR plasmid at 24 hours following transfection. The values in each quadrant represent the percentage of total gated live CD3^{+}CD8^{+}cells.

Supplementary Table 1. List of T-cell lines stimulated and expanded through various approaches. *

	LCL	TPA/NaB	moDC
T_{LCL}	+	-	-
T_{lyLCL}	+	+	-
T_{DC+LCL}	+	-	+
$T_{DC+lyLCL}$	+	+	+

LCL, lymphoblastoid cell line; TPA, 12- tetradecanoylphorbol 13-acetate; NaB, sodium butyrate; moDC, monocyte derived dendritic cells.

*All EBV enriched T-cell lines were isolated and expanded from 6 donors (*n*=6). Four EBV-specific T-cell lines were generated from each donor. The T_{LCL} line is produced by stimulating with LCLs, T_{lyLCL} line is produced by stimulating with LCLs that were pre-treated with TPA and NaB. T_{DC+LCL} and $T_{DC+lyLCL}$ lines were produced in the presence of autologous moDC plus LCLs or TPA/NaB treated LCLs, respectively.

Supplementary Table 2. Overlapping minigenes of EBNA3C and EBNA3B.

Truncated ORF co-ordinates (amino acid)					
EBNA3C	1-300	125-475	451-750	601-900	751-992
EBNA3B	1-300	125-475	451-750	601-900	751-938

Supplementary Table 3. *In silico* predicted epitopes in the first 125 amino acids of EBNA3C.

Allele	Start	End	Length	Peptide	Method	Percentile rank
HLA-B*57:01	60	69	10	QSRGDENRGW	Consensus (ann/smm)	0.75
HLA-B*57:01	62	69	8	RGDENRGW	ann	0.5
HLA-B*57:01	51	58	8	YSRDQQPW	ann	0.1

The predictions of HLA binding were performed with allele-specific algorithms using the IEDB recommended consensus method

Supplementary Table 4. List of EBV positive donors used in this study and their available HLA typing information.

Donor	HLA-A1	HLA-A2	HLA-B1	HLA-B2	HLA-DRB11	HLA-DRB12	*EBV
1	02:AMAY˜	NA	57:01	35:03	13:03	14:JCJ#	Positive
2	31:01	32:01	57:01	35:03	NA	NA	Positive
3	29:02	68:02	57:01	44:03	NA	NA	Positive
4	01	02	57:01	07:02	NA	NA	Positive
5	01CRY^	3:01	57:01	07:02	NA	NA	Positive
6	01	3:01	57:01	14:02	NA	NA	Positive
7	01	32	57:01	40	NA	NA	Positive
8	01CRY^	02BDDU&	57:01	44WRJ$	NA	NA	Positive
9	01	02	57:01	07:02	NA	NA	Positive

*All donors were tested with EBV epitope (VSFIEFVGW) to confirm the donors were positive to EBV; NA-information not available. The old allele nomenclature

˜ HLA-A*02:AMAY is equivalent to A*02:01/A*02:01L/A*02:09/A*02:43N/A*02:66;

^ HLA-A*01CRY to A*01:01/A*01:04N;

& HLA-A*02BDDU to A* 02:01/A*02:01L/A*02:09/A*02:43N/A*02:66/A*02:75;

$ HLA-B*44WRJ to 44:02/B*44:02S/B*44:19N/B*44:27; and

HLA-DRB1*14:JCJ to DRB1*14:01/DRB1*14:39.

Supplementary Table 5. Primers used to construct pAR100 and pAR101 plasmids.

Primer name	Primer sequence (5' - 3')
CD8_F	aagtccACCGGTGCCACCATGGCCTTACCAGTGACCG
CD8_R	atcgatCTTAAGGACGTATCTCGCCGAAAGG
MCS-I-F	CTAGCCTTAAGGGGCCCGGCGCGCCCGTACGGTATACTCGCGACTGCAGCCGCGGG
MCS-I-R	CTAGCCCGCGGCTGCAGTCGCGAGTATACCGTACGGGCGCGCCGGGCCCCTTAAGG
MCS-II-F	AATTCGGTACCTCTAGAGGATCCGTCGACG
MCS-II-R	AATTCGTCGACGGATCCTCTAGAGGTACCG
attR1-KpnI	taagcaGGTACCACAAGTTTGTACAAAAAAGCTGAAC
attR2-EcoRV	tgcttaGATATCACCACTTTGTACAAGAAAGCTGAAC
