# Supplementary material for: Abacavir inhibits but does not cause self-reactivity to HLA-B*57:01-restricted EBV specific T cell receptors
Source: Commun Biol. 2022 Feb 16;5:133. doi: 10.1038/s42003-022-03058-9 (PMC8850454; doi:10.1038/s42003-022-03058-9)
Supplement: Supplementary file 4 — Reporting Summary [file 42003_2022_3058_MOESM4_ESM.pdf]

nature research

Corresponding author(s): Anuradha Sooda

Last updated by author(s): Dec 21, 2021

Reporting Summary

Nature Research wishes to improve the reproducibility of the work that we publish. This form provides structure for consistency and transparency in reporting. For further information on Nature Research policies, see our Editorial Policies and the Editorial Policy Checklist.

Statistics

For all statistical analyses, confirm that the following items are present in the figure legend, table legend, main text, or Methods section.

n/a	Confirmed	
☒	☐	The exact sample size (n) for each experimental group/condition, given as a discrete number and unit of measurement
☒	☐	A statement on whether measurements were taken from distinct samples or whether the same sample was measured repeatedly
☒	☐	The statistical test(s) used AND whether they are one- or two-sided<br>*Only common tests should be described solely by name; describe more complex techniques in the Methods section.*
☒	☐	A description of all covariates tested
☒	☐	A description of any assumptions or corrections, such as tests of normality and adjustment for multiple comparisons
☒	☐	A full description of the statistical parameters including central tendency (e.g. means) or other basic estimates (e.g. regression coefficient) AND variation (e.g. standard deviation) or associated estimates of uncertainty (e.g. confidence intervals)
☒	☐	For null hypothesis testing, the test statistic (e.g. F, t, r) with confidence intervals, effect sizes, degrees of freedom and P value noted<br>*Give P values as exact values whenever suitable.*
☒	☐	For Bayesian analysis, information on the choice of priors and Markov chain Monte Carlo settings
☒	☐	For hierarchical and complex designs, identification of the appropriate level for tests and full reporting of outcomes
☒	☐	Estimates of effect sizes (e.g. Cohen's d, Pearson's r), indicating how they were calculated

Our web collection on statistics for biologists contains articles on many of the points above.

Software and code

Policy information about availability of computer code

Data collection	Absorbance was read at 450 nm wavelength using a DTX 880 multimode detector (Beckman Coulter).<br>ELISpot data was counted with an automated imaging device, AID ELISpot analyser (AID GmbH)<br>Flow cytometry data was collected on a Beckman Coulter Gallios (Beckman Coulter).<br>Cell sorting was done using the FACSAria III cell sorter(BD Biosciences)<br>Luciferase reporter was measured on a DTX 880 multimode detector (Beckman Coulter).
Data analysis	Data analysis was performed in GraphPad Prism® Version 9.1.0 Software.<br>Flow data analysis was performed using Kaluza software (Beckman Coulter).<br>TCR sequencing data was analysed using in-house Visual Genome Analysis Studio software package.

For manuscripts utilizing custom algorithms or software that are central to the research but not yet described in published literature, software must be made available to editors and reviewers. We strongly encourage code deposition in a community repository (e.g. GitHub). See the Nature Research guidelines for submitting code & software for further information.

Data

Policy information about availability of data

All manuscripts must include a data availability statement. This statement should provide the following information, where applicable:

- Accession codes, unique identifiers, or web links for publicly available datasets
- A list of figures that have associated raw data
- A description of any restrictions on data availability

Raw TCR sequencing data are available on the SRA database, accession number PRJNA706784. Figure 8 is associated with the analyzed data

Plasmid sequences were submitted to Addgene repository (plasmid#180377; plasmid#180378). Supplementary Fig. 1-2 are associated with it.

Field-specific reporting

Please select the one below that is the best fit for your research. If you are not sure, read the appropriate sections before making your selection.

- [x] Life sciences
- [ ] Behavioural & social sciences
- [ ] Ecological, evolutionary & environmental sciences

For a reference copy of the document with all sections, see nature.com/documents/nr-reporting-summary-flat.pdf

Life sciences study design

All studies must disclose on these points even when the disclosure is negative.

Sample size	Sample size for EBV ORF screening was 6 (n=6) and to screen epitope specific responses in the blood we used 9 (n=9)
Data exclusions	No data were excluded
Replication	All the experiments were conducted in duplicates and all the replications were successful
Randomization	Randomization was not relevant to this study as we were testing CD8 T cell responses in HLA-B*57:01 donors.
Blinding	Blinding was not relevant to our study because we tested a single group of participants.

Reporting for specific materials, systems and methods

We require information from authors about some types of materials, experimental systems and methods used in many studies. Here, indicate whether each material, system or method listed is relevant to your study. If you are not sure if a list item applies to your research, read the appropriate section before selecting a response.

Materials & experimental systems

n/a	Involved in the study	
[ ]	[x]	Antibodies
[ ]	[x]	Eukaryotic cell lines
[x]	[ ]	Palaeontology and archaeology
[x]	[ ]	Animals and other organisms
[ ]	[x]	Human research participants
[x]	[ ]	Clinical data
[x]	[ ]	Dual use research of concern

Methods

n/a	Involved in the study	
[x]	[ ]	ChIP-seq
[ ]	[x]	Flow cytometry
[x]	[ ]	MRI-based neuroimaging

Antibodies

Antibodies used

7-aminoactinomycin D (supplier: BD Biosciences; cat# 51-68981E(sold as 559925; lot# 6084701),
anti-CD3-PE (clone UCHT1; supplier: BD Biosciences; cat# 555333; lot# 6210926),
anti-CD8-FITC (clone 3B5; supplier: Invitrogen; cat# MHCD0801; lot# 1661186A),
and anti-CD137-Allophycocyanin (clone 4B4-1; supplier: BD Biosciences; cat# 550890; lot# 7118688)
Fixable viability stain 620 (supplier: BD Biosciences; cat# 564996; lot# 7179617)
CD3-Alexa Flour 700 (clone UCHT1; supplier: BD Biosciences; cat# 557943; lot# 7117648)
anti-CD8a Allophycocyanin-Fire 750 (clone RPA-T8, supplier: BioLegend, cat# 301065, lot# B23387)

Validation

Antibodies were validated with beads

Eukaryotic cell lines

Policy information about cell lines

Cell line source(s)

K562-HLA null parental lines were obtained from Dr. Yvonne Zoet (Leiden University) and K562-B*57:01 single antigen lines were produced in our laboratory by Dr. Dr. Coral-Ann Almeida (Institute for Immunology and Infectious Diseases).
Cos7 cell line was obtained from Deparment of Microbiology (Royal Perth Hospital), which were obtained from ATCC.
Jurkat clone E6-1 was obtained from National Institute of Health.
B95-8 cell line was obtained from Deparment of Microbiology (Royal Perth Hospital)

Authentication

K562-HLA null parental lines were authenticated by HLA typing in ASHI accredited lab (Institute for Immunology and Infectious Diseases, Murdoch University, Australia).

K562-B*57:01 was confirmed by the expression of B57 on flow cytometry using anti-B17 (B57/58) antibody - cat# 0196HA, lot#001(One Lambda) paired with goat anti-mouse IgM FITC - cat# F9259, lot# SLBT0614 (Sigma-Aldrich)
Jurkat clone E6-1 was confirmed by HLA typing.
B95-8 cell line confirmed by the production of EBV.

Mycoplasma contamination	Continuous cell lines were Mycoplasma negative
Commonly misidentified lines (See ICLAC register)	No commonly misidentified lines were used

Human research participants

Policy information about studies involving human research participants

Population characteristics	Healthy donors carrying the HLA-B*57:01 allele and seropositive for EBV were used in the study.
Recruitment	All the donors used in the study were recruited at the Australian Bone Marrow Donor Registry
Ethics oversight	Ethics approval for the conduct of this research project was obtained from Murdoch University Human Research Ethics Committee. (2017/246)

Note that full information on the approval of the study protocol must also be provided in the manuscript.

Flow Cytometry

Plots

Confirm that:

- [x] The axis labels state the marker and fluorochrome used (e.g. CD4-FITC).
- [x] The axis scales are clearly visible. Include numbers along axes only for bottom left plot of group (a 'group' is an analysis of identical markers).
- [x] All plots are contour plots with outliers or pseudocolor plots.
- [x] A numerical value for number of cells or percentage (with statistics) is provided.

Methodology

Sample preparation	After antibody staining, the cells were washed 3 times and strained using a polystyrene tube with a cell strainer cap before cell sorting
Instrument	FACSAria II (BD Biosciences) for cell sorting and Beckman Coulter Gallios (Beckman Coulter) for flow cytometry
Software	The data was analysed using Kaluza software (Beckman Coulter)
Cell population abundance	The post-sort fractions of EBV-specific T cells and non-EBV specific T cells (control) were expanded under similar conditions and both the fractions were tested through intra-cellular staining for the abundance of the EBV-specific T cells
Gating strategy	The gating strategy involved first identifying the lymphocyte population by forward scatter and side scatter. Live CD3+T cells were gated and then the cells were gated on CD8+ .

- [x] Tick this box to confirm that a figure exemplifying the gating strategy is provided in the Supplementary Information.
